# Supplementary figures and images for: Pulmonary vascular dysfunction among people aged over 65 years in the community in the Atherosclerosis Risk In Communities (ARIC) Study: A cross-sectional analysis
Source: PLoS Med. 2020 Oct 15;17(10):e1003361. doi: 10.1371/journal.pmed.1003361 (PMC7561082; doi:10.1371/journal.pmed.1003361)

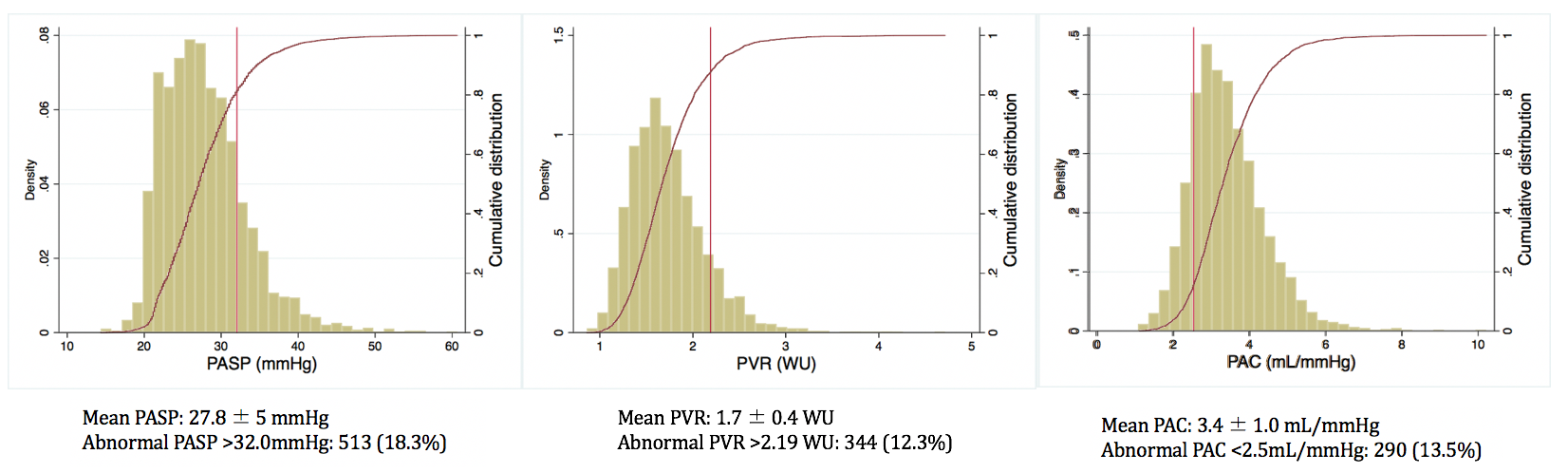

Supplement: S1 Fig — Density and cumulative distributions are presented. Dashed lines represent the reference limit derived based on the 10th percentile of the low-risk subset of participants for PASP and PVR. For PAC, the 90th percentile of the low-risk subset of participants were used as reference limit. PAC, pulmonary arterial compliance; PASP, pulmonary artery systolic pressure; PVR, pulmonary vascular resistance. (TIF) [file pmed.1003361.s002.tif]

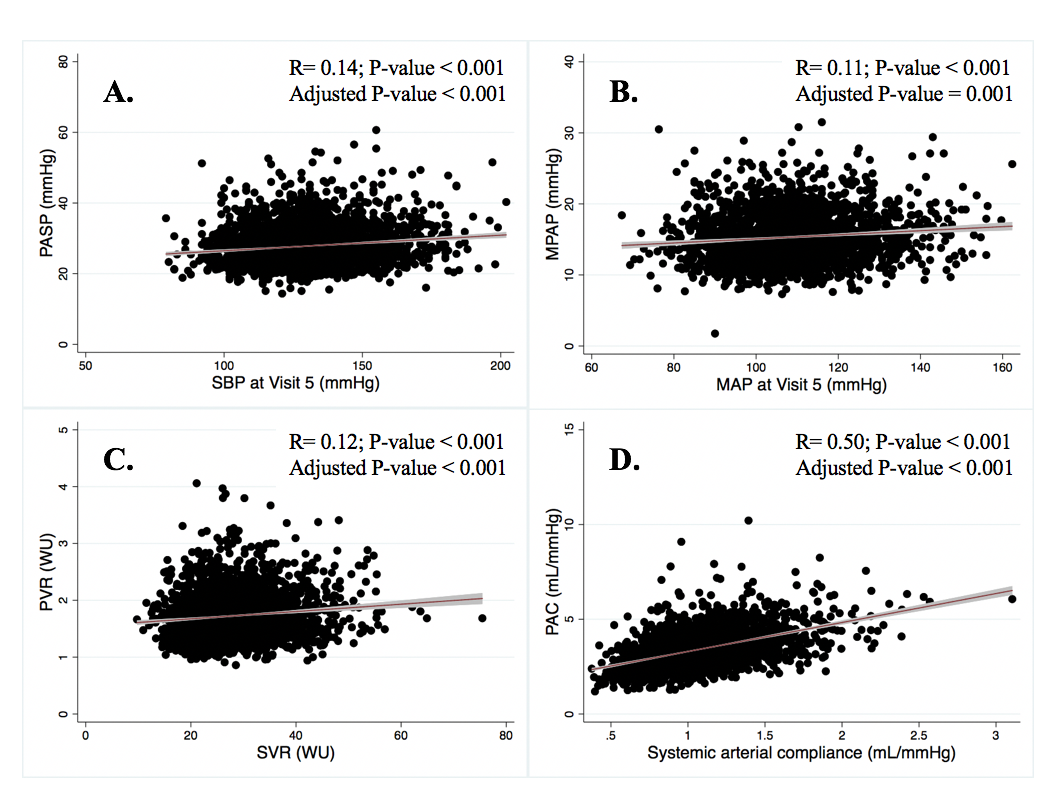

Supplement: S2 Fig — (A) PASP and SBP; (B) MPAP and MAP; (C) PVR and SVR; and (D) PAC and SAC. p-Values were derived from multivariable regression model adjusted for age, sex, race, visit center, BMI, hypertension, diabetes, and LV measures (LVEF, LAVi, LVMi, and septal E/E’). BMI, body mass index; LAVi, left atrial volume index; LV, left ventricular; LVEF, LV ejection fraction; LVMi, LV mass index; MAP, mean arterial pressure; MPAP, mean pulmonary arterial pressure; PAC, pulmonary arterial compliance; PASP, pulmonary artery systolic pressure; PVR, pulmonary vascular resistance; SAC, systemic arterial compliance; SVR, systemic vascular resistance. (TIF) [file pmed.1003361.s003.tif]

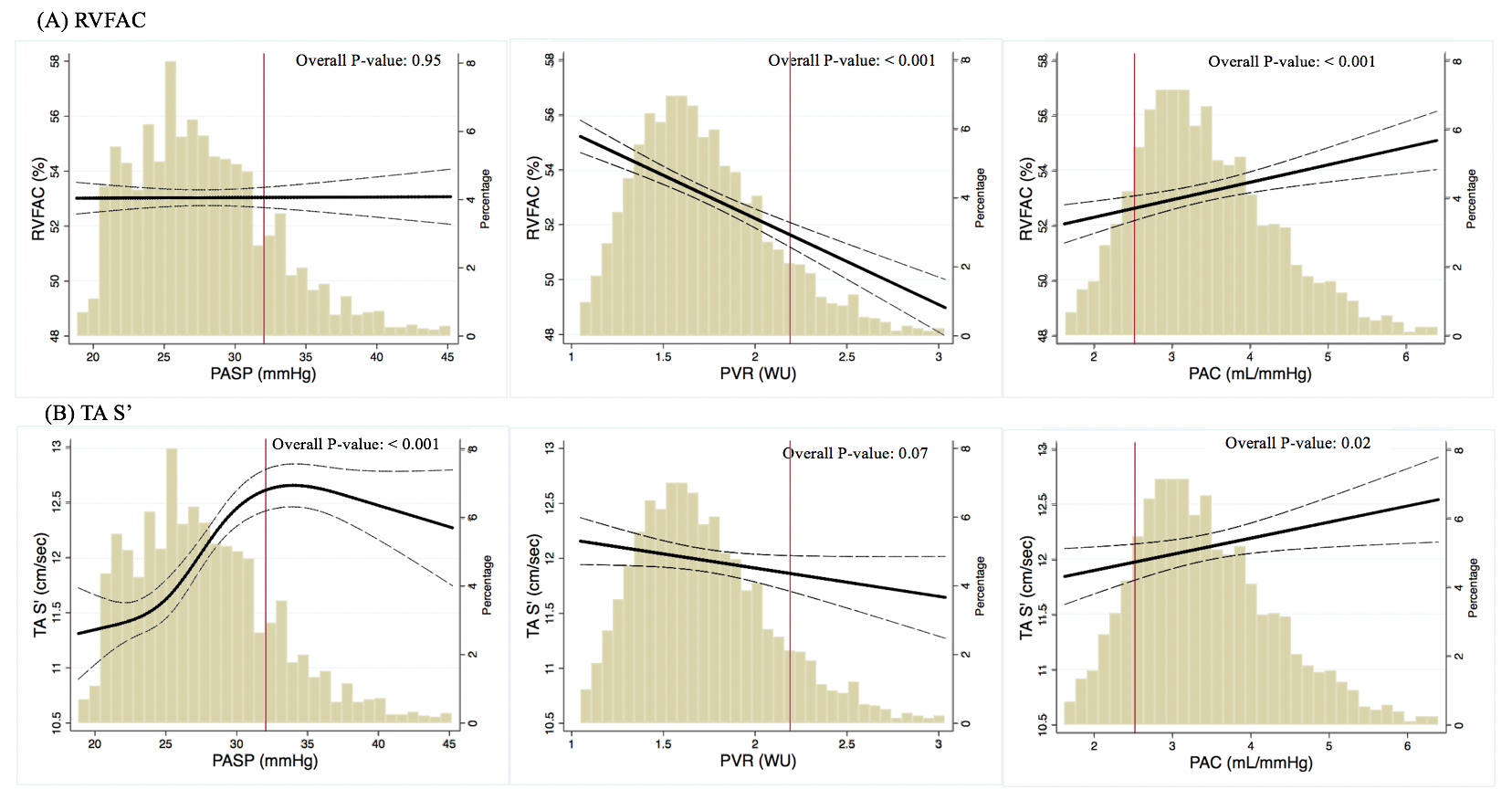

Supplement: S3 Fig — p-Values were derived from cubic spline regression model adjusted for age, sex, race, visit center, BMI, hypertension, diabetes, LVEF, LAVi, LVMi, and septal E/e’. BMI, body mass index; LAVi, left atrial volume index; LVEF, left ventricular ejection fraction; LVMi, left ventricular mass index; PAC, pulmonary arterial compliance; PASP, pulmonary artery systolic pressure; PVR, pulmonary vascular resistance; RV, right ventricular; RVFAC, RV fractional area change; TA s', tricuspid annulus. (TIF) [file pmed.1003361.s004.tif]

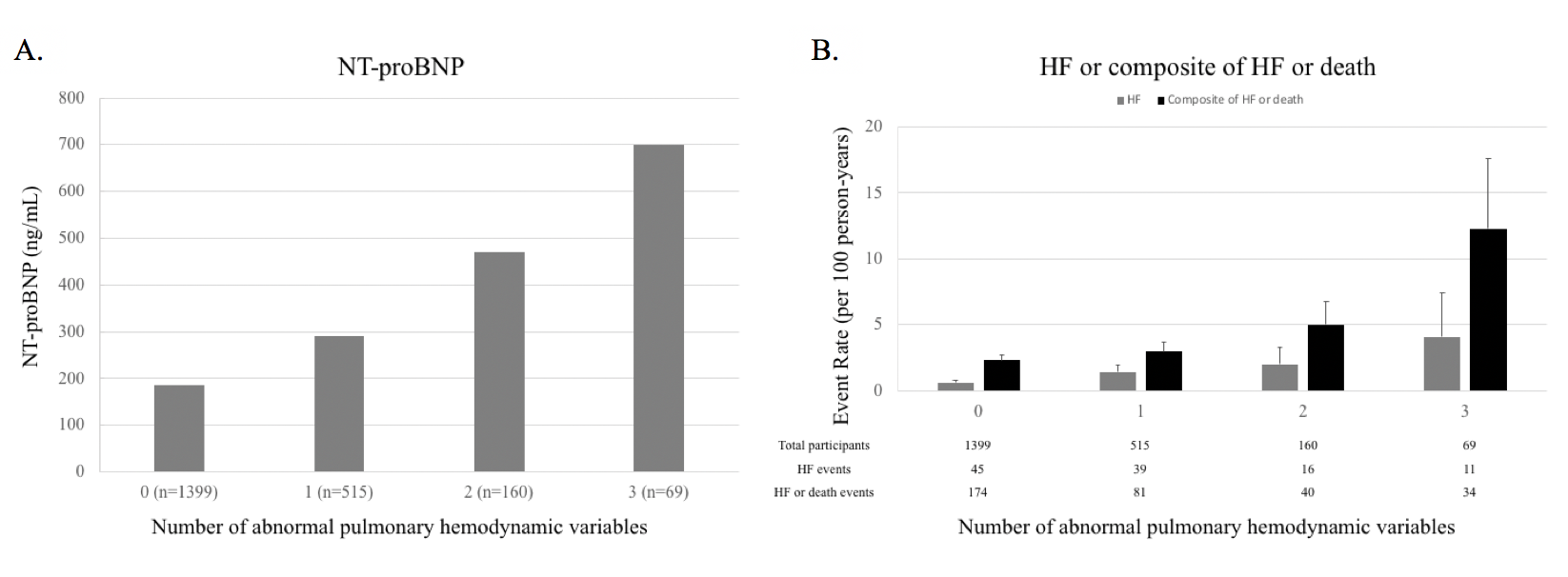

Supplement: S4 Fig — Event rate in per 100 person-years and error bars presenting the upper limit of 95% confidence interval. HF, heart failure; NT-proBNP, N-terminal prohormone brain natriuretic peptide. (TIF) [file pmed.1003361.s005.tif]
