# Supplementary material for: Pulmonary vascular dysfunction among people aged over 65 years in the community in the Atherosclerosis Risk In Communities (ARIC) Study: A cross-sectional analysis
Source: PLoS Med. 2020 Oct 15;17(10):e1003361. doi: 10.1371/journal.pmed.1003361 (PMC7561082; doi:10.1371/journal.pmed.1003361)
Supplement: S1 Table — All continuous variables are described as mean ± SD. Nonparametric values are presented with median and interquartile range in square brackets. p-Values are derived from ANOVA for continuous variables, Pearson chi-squared test for binary and categorical variables, and Kruskal-Wallis test for nonparametric continuous variables. Afib, atrial fibrillation; BMI, body mass index; CAD, coronary artery disease; CKD, chronic kidney disease; DBP, diastolic blood pressure; eGFR, estimated glomerular filtration rate; HR, heart rate; hs-CRP, high-sensitivity C-reactive protein; hs-TnT, high-sensitivity troponin T; LAVi, left atrial volume index; LVEDD, left ventricular end-diastolic diameter; LVEF, left ventricular ejection fraction; LVM, left ventricular mass; LVMi, LVM index; MI, myocardial infarction; MWT, mean wall thickness; PAD, peripheral artery disease; RVFAC, right ventricle fractional area change; RWT, relative wall thickness; SBP, systolic blood pressure; TA S', tricuspid annulus. (DOCX) [file pmed.1003361.s006.docx]

**S1 Table. Clinical and echocardiographic characteristics of participants included in the study versus those not including in the study.**

|  | Excluded | Included | P-value |
| --- | --- | --- | --- |
|  | (n=12,982) | (n=2,810) |  |
| **Demographics** |  |  |  |
| Age, years | 76.2 ± 5.2 | 76.2 ± 4.8 | 0.77 |
| Male sex, % | 6113 (47 %) | 969 (34 %) | < 0.001 |
| Black, % | 3666 (28 %) | 600 (21 %) | 0.06 |
| **Field Center** |  |  | 0.28 |
| Forsyth County, North Carolina | 3229 (24.9%) | 806 (28.7%) |  |
| Jackson, Mississippi | 3189 (24.6%) | 539 (19.2%) |  |
| Minneapolis, Minnesota | 3366 (25.9%) | 643 (22.9%) |  |
| Washington County, Maryland | 3198 (24.6%) | 822 (29.3%) |  |
| **Comorbidities** |  |  |  |
| Hypertension, % | 3244 (87 %) | 2255 (80 %) | < 0.001 |
| Diabetes, % | 1623 (44 %) | 892 (32 %) | < 0.001 |
| Obesity, % | 1069 (40 %) | 815 (29 %) | < 0.001 |
| Metabolic Syndrome, % | 1743 (66 %) | 1510 (55 %) | < 0.001 |
| CKD, % | 1130 (31 %) | 744 (27 %) | < 0.001 |
| Past history of smoking, % | 2397 (64 %) | 1618 (58 %) | < 0.001 |
| Current smoker, % | 213 (6 %) | 150 (6 %) | 0.24 |
| **Prevalent cardiovascular conditions** | |  |  |
| CAD, % | 502 (19 %) | 300 (11 %) | < 0.001 |
| Previous MI, % | 473 (14 %) | 96 (4 %) | < 0.001 |
| PAD, % | 387 (14 %) | 378 (13 %) | 0.31 |
| Previous stroke, % | 197 (5 %) | 73 (3 %) | < 0.001 |
| Previous Afib, % | 342 (9 %) | 155 (6 %) | < 0.001 |
| **Physical exam** |  |  |  |
| BMI, Kg/m^2^ | 29.5 ± 6.1 | 27.8 ± 5.3 | < 0.001 |
| SBP, mmHg | 131 ± 19 | 130 ± 18 | 0.06 |
| DBP, mmHg | 67 ± 11 | 67 ± 10 | 0.42 |
| HR, bpm | 63 ± 11 | 62 ± 10 | < 0.001 |
| **Laboratory values** |  |  |  |
| eGFR, ml/min/1.73m^2^ | 68.2 ± 18.2 | 70.4 ± 16.7 | < 0.00 |
| hs-TnT, ng/mL | 0.012 [0.008, 0.018] | 0.010 [0.007, 0.015] | < 0.001 |
| hs-CRP, mg/L | 2.2 [1.0, 4.8] | 1.9 [0.9, 4.0] | < 0.001 |
| **Echocardiographic data** |  |  |  |
| *LV Structure* |  |  |  |
| LVEDD, cm | 4.44 ± 0.52 | 4.33 ± 0.47 | < 0.001 |
| MWT, cm | 1.01 ± 0.15 | 0.97 ± 0.13 | < 0.001 |
| LVMi, mg/m^2^ | 80 ± 20 | 77 ± 18 | < 0.001 |
| RWT | 0.4 ± 0.1 | 0.4 ± 0.1 | < 0.001 |
| *LV Systolic Function* |  |  |  |
| LVEF, % | 65.0 ± 6.5 | 66.1 ± 5.8 | < 0.001 |
| LV longitudinal strain, % | -17.4 ± 2.8 | -18.3 ± 2.4 | < 0.001 |
| *LV Diastolic Function* |  |  |  |
| TDI E' septal, cm/s | 5.6 ± 1.4 | 5.8 ± 1.5 | < 0.001 |
| E/E' septal | 12.2 ± 4.4 | 12.3 ± 4.1 | 0.53 |
| LAVi, mL/m^2^ | 24.8 ± 8.0 | 26.3 ± 9.0 | < 0.001 |
| *RV Function* |  |  |  |
| RVFAC, % | 52 ± 7 | 53 ± 8 | < 0.001 |
| TA S', cm/s | 11.3 ± 2.9 | 12.0 ± 2.9 | < 0.001 |

Legend: CKD, chronic kidney disease; CAD, coronary artery disease; MI, myocardial infarction; PAD, peripheral artery disease; Afib, atrial fibrillation; BMI, body mass indexed; SBP, systolic blood pressure; DBP, diastolic blood pressure; HR, heart rate; eGFR, estimated glomerular filtration rate; hs-TnT, high-sensitivity troponin T; hs-CRP, high-sensitivity C-reactive protein; LVEDD, left ventricular end-diastolic diameter; MWT, mean wall thickness; LVM, left ventricular mass; LVMi, left ventricular mass indexed; RWT, relative wall thickness; LVEF, left ventricular ejection fraction; LAVi, left atrial volume indexed; RVFAC, right ventricle fractional area change; TA S', tricuspid annulus. All continuous variables are described in mean ± standard deviation. Non-parametric values are presented with median and inter quartile range in square brackets. P-values are derived from ANOVA for continuous variables, Pearson chi-squared test for binary and categorical variables, and Kruskal-Wallis test for non-parametric continuous variables.
