# Supplementary material for: Pulmonary vascular dysfunction among people aged over 65 years in the community in the Atherosclerosis Risk In Communities (ARIC) Study: A cross-sectional analysis
Source: PLoS Med. 2020 Oct 15;17(10):e1003361. doi: 10.1371/journal.pmed.1003361 (PMC7561082; doi:10.1371/journal.pmed.1003361)
Supplement: S2 Table — All continuous variables are described as mean ± SD. Nonparametric values are presented with median and interquartile range in square brackets. p-Values are derived from ANOVA for continuous variables, Pearson chi-squared test for binary and categorical variables, and Kruskal-Wallis test for nonparametric continuous variables. Afib, atrial fibrillation; BMI, body mass index; CAD, coronary artery disease; CKD, chronic kidney disease; DBP, diastolic blood pressure; eGFR, estimated glomerular filtration rate; HR, heart rate; hs-CRP, high-sensitivity C-reactive protein; hs-TnT, high-sensitivity troponin T; MI, myocardial infarction; PAD, peripheral artery disease; SBP, systolic blood pressure. (DOCX) [file pmed.1003361.s007.docx]

**S2 Table. Clinical and echocardiographic characteristics of study participants in low-risk subgroup compared to those not in the low-risk subgroup.**

|  | Non-low-risk | Low-risk reference | P-value |
| --- | --- | --- | --- |
|  | Subgroup (n=2,557) | Subgroup (n=253) |  |
| **Demographics** |  |  |  |
| Age, years | 76.4 ± 5.2 | 74.7 ± 4.6 | < 0.001 |
| Male sex, % | 895 (35 %) | 74 (29 %) | 0.07 |
| Black, % | 579 (23 %) | 21 (8 %) | < 0.001 |
| Field Center |  |  | 0.17 |
| Forsyth County, North Carolina | 713 (27.9%) | 93 (36.8%) |  |
| Jackson, Mississippi | 520 (20.3%) | 19 (7.5 %) |  |
| Minneapolis, Minnesota | 565 (22.1%) | 78 (30.8%) |  |
| Washington County, Maryland | 759 (29.7%) | 63 (24.9%) |  |
| **Comorbidities** |  |  |  |
| Hypertension, % | 2255 (88 %) | 0 (0 %) | < 0.001 |
| Diabetes, % | 892 (35 %) | 0 (0 %) | < 0.001 |
| Obesity, % | 815 (32 %) | 0 (0 %) | < 0.001 |
| Metabolic Syndrome, % | 1476 (59 %) | 34 (13 %) | < 0.001 |
| CKD, % | 744 (29 %) | 0 (0 %) | < 0.001 |
| Past history of smoking, % | 1484 (58 %) | 134 (53 %) | 0.12 |
| Current smoker, % | 150 (6 %) | 0 (0 %) | < 0.001 |
| **Prevalent cardiovascular conditions** | |  |  |
| CAD, % | 300 (12 %) | 0 (0 %) | N/A |
| Previous MI, % | 96 (4 %) | 0 (0 %) | N/A |
| PAD, % | 371 (15 %) | 7 (3 %) | < 0.001 |
| Previous stroke, % | 73 (3 %) | 0 (0 %) | N/A |
| Previous Afib, % | 155 (6 %) | 0 (0 %) | N/A |
| **Physical exam** |  |  |  |
| BMI, Kg/m^2^ | 28.1 ± 5.3 | 24.6 ± 2.8 | < 0.001 |
| SBP, mmHg | 131 ± 18 | 120 ± 11 | < 0.001 |
| DBP, mmHg | 67 ± 11 | 64 ± 8 | < 0.001 |
| HR, bpm | 62 ± 10 | 60 ± 8 | 0.02 |
| **Laboratory values** |  |  |  |
| eGFR, ml/min/1.73m^2^ | 69.6 ± 17.1 | 77.5 ± 10.1 | < 0.001 |
| NT-pro-BNP, ng/mL | 140.9 [72.1, 266.5] | 96.7 [55.5, 167.6] | < 0.001 |
| hs-TnT, ng/mL | 0.010 [0.007, 0.015] | 0.007 [0.005, 0.009] | < 0.001 |
| hs-CRP, mg/L | 1.9 [0.9, 4.1] | 1.3 [0.7, 2.6] | < 0.001 |

Legend: CKD, chronic kidney disease; CAD, coronary artery disease; MI, myocardial infarction; PAD, peripheral artery disease; Afib, atrial fibrillation; BMI, body mass indexed; SBP, systolic blood pressure; DBP, diastolic blood pressure; HR, heart rate; eGFR, estimated glomerular filtration rate; hs-TnT, high-sensitivity troponin T; hs-CRP, high-sensitivity C-reactive protein. All continuous variables are described in mean ± standard deviation. Non-parametric values are presented with median and inter quartile range in square brackets.

P-values are derived from ANOVA for continuous variables, Pearson chi-squared test for binary and categorical variables, and Kruskal-Wallis test for non-parametric continuous variables.
