# Supplementary material for: Pulmonary vascular dysfunction among people aged over 65 years in the community in the Atherosclerosis Risk In Communities (ARIC) Study: A cross-sectional analysis
Source: PLoS Med. 2020 Oct 15;17(10):e1003361. doi: 10.1371/journal.pmed.1003361 (PMC7561082; doi:10.1371/journal.pmed.1003361)
Supplement: S3 Table — The 10th, 50th, and 90th percentile values with associated 95% confidence intervals derived from quantile regression models in the low-risk reference subgroup overall and separately by sex. PAC, pulmonary arterial compliance; PAP, pulmonary arterial pressure; PASP, pulmonary arterial systolic pressure; PVR, pulmonary vascular resistance; TR, tricuspid regurgitation. (DOCX) [file pmed.1003361.s008.docx]

**S3 Table. Percentile limits for pulmonary hemodynamic measures among the low risk reference subgroup (n=253).**

|  | 10th percentile  [95% CI] | 50th percentile  [95% CI] | 90th percentile [95% CI] |
| --- | --- | --- | --- |
| **TR velocity, m/s** |  |  |  |
| Overall | 2.02 [1.99-2.05] | 2.29 [2.25-2.33] | 2.60 [2.56-2.64] |
| Female | 2.01 [1.98-2.04] | 2.29 [2.24-2.33] | 2.62 [2.58-2.66] |
| Male | 2.02 [1.97-2.07] | 2.29 [2.22-2.36] | 2.57 [2.50-2.64] |
| **PASP, mmHg** |  |  |  |
| Overall | 21.3 [20.8-21.8] | 26.0 [25.3-26.7] | 32.0 [31.1-33.0] |
| Female | 21.2 [20.7-21.7] | 26.0 [25.2-26.8] | 32.5 [31.6-33.4] |
| Male | 21.3 [20.6-22.1] | 26.0 [24.7-27.2] | 31.4 [30.0-32.8] |
| **Mean PAP, mmHg** |  |  |  |
| Overall | 10.7 [10.1-11.3] | 13.9 [13.5-14.3] | 17.7 [16.6-18.8] |
| Female | 10.8 [10.1-11.5] | 13.8 [13.3-14.3] | 18.4 [17.2-19.6] |
| Male | 10.1 [9.0-11.2] | 13.9 [13.1-14.7] | 17.4 [15.6-19.2] |
| **PAC, mL/mmHg** |  |  |  |
| Overall | 2.5 [2.3-2.7] | 3.3 [3.2-3.5] | 4.6 [4.3-4.9] |
| Female | 2.4 [2.2-2.5] | 3.3 [3.1-3.5] | 4.2 [3.9-4.5] |
| Male | 2.7 [2.4-3.0] | 3.6 [3.3-3.8] | 4.8 [4.4-5.3] |
| **PVR, WU** |  |  |  |
| Overall | 1.31 [1.26-1.36] | 1.71 [1.66-1.76] | 2.19 [2.07-2.31] |
| Female | 1.32 [1.26-1.38] | 1.67 [1.60-1.74] | 2.13 [1.99-2.27] |
| Male | 1.23 [1.14-1.32] | 1.76 [1.66-1.86] | 2.28 [2.06-2.50] |

Legend: TR, tricuspid regurgitation; PASP, pulmonary arterial systolic pressure; PAP, pulmonary arterial pressure; PAC, pulmonary arterial compliance; PVR, pulmonary vascular resistance. 10th, 50th and 90th-percentile values with associated 95% confidence intervals derived from quantile regression models in the low risk reference subgroup overall and separately by sex.
