# Supplementary material for: Pulmonary vascular dysfunction among people aged over 65 years in the community in the Atherosclerosis Risk In Communities (ARIC) Study: A cross-sectional analysis
Source: PLoS Med. 2020 Oct 15;17(10):e1003361. doi: 10.1371/journal.pmed.1003361 (PMC7561082; doi:10.1371/journal.pmed.1003361)
Supplement: S4 Table — Estimates for COPD and restrictive lung disease as measures of pulmonary dysfunction are provided separately. Logistic regression models are used to estimate the odds ratio and p-values of each pulmonary vasculature dysfunction for having abnormal pulmonary measures. The model contains age, sex, race, visit center, LHD, COPD, restrictive lung disease, and prior VTE. COPD, chronic obstructive pulmonary disease; LHD, left heart dysfunction; PAR, populational attributable risk; VTE, venous thromboembolism. (DOCX) [file pmed.1003361.s009.docx]

**S4 Table.** **Prevalence of left heart dysfunction, pulmonary dysfunction (COPD and restrictive lung disease), and VTE, and their association with measures of pulmonary vascular dysfunction.**

|  | N abnormal | OR [95%CI] | P value | PAR [95%CI] |
| --- | --- | --- | --- | --- |
| **Abnormal PASP** | Total n=2810 |  |  |  |
| LHD | 798 (28%) | 2.11 [1.72-2.58] | < 0.001 | 18.9 [13.3-24.1] |
| COPD | 780 (28%) | 1.27 [1.03-1.58] | 0.027 | 5.5 [0.4-10.4] |
| Restrictive lung disease | 329 (12%) | 2.07 [1.58-2.71] | < 0.001 | 8.0 [4.7-11.2] |
| Prior VTE | 76 (3%) | 1.17 [0.67-2.05] | 0.57 | 3.9 [-1.0, 1.8] [NS] |
| **Abnormal PVR** | Total n=2798 |  |  |  |
| LHD | 793 (28%) | 1.12 [0.88-1.44] | 0.36 | 3.2 [-3.9, 9.8] [NS] |
| COPD | 775 (28%) | 1.01 [0.78-1.32] | 0.91 | 0.3 [-6.0, 6.3] [NS] |
| Restrictive lung disease | 327 (12%) | 1.82 [1.31-2.54] | < 0.001 | 6.2 [2.3, 10.1] |
| Prior VTE | 76 (3%) | 0.93 [0.46-1.88] | 0.85 | -0.2 [-1.8, 1.5] [NS] |
| **Abnormal PAC** | Total n=2149 |  |  |  |
| LHD | 633 (29%) | 1.47 [1.12-1.92] | 0.005 | 10.4 [2.6-17.5] |
| COPD | 602 (28%) | 1.40 [1.07-1.92] | 0.015 | 8.7 [1.2-15.5] |
| Restrictive lung disease | 249 (12%) | 1.96 [1.12-2.77] | 0.001 | 7.9 [3.2-12.3] |
| Prior VTE | 62 (3%) | 0.61 [0.27-1.38] | 0.23 | -1.2 [-2.9, 0.5] [NS] |

Legend: LHD, left heart dysfunction; COPD, chronic obstructive pulmonary disease; VTE, venous thromboembolism; PAR, population attributable risk. Estimates for COPD and restrictive lung disease as measures of pulmonary dysfunction are provided separately. Logistic regression models are used to estimate the odds ratio and p-values of each pulmonary vasculature dysfunction for having abnormal pulmonary measures. The model contains age, sex, race, visit center, LHD, COPD, restrictive lung disease, and prior VTE.
