# Supplementary material for: Pulmonary vascular dysfunction among people aged over 65 years in the community in the Atherosclerosis Risk In Communities (ARIC) Study: A cross-sectional analysis
Source: PLoS Med. 2020 Oct 15;17(10):e1003361. doi: 10.1371/journal.pmed.1003361 (PMC7561082; doi:10.1371/journal.pmed.1003361)
Supplement: S5 Table — Odds values and p-values are derived from multivariable logistic models containing age, sex, race, visit center, LHD, pulmonary dysfunction, and prior VTE. LHD, left heart disease; NS, not significant; PAR, population attributable risk; PASP, pulmonary artery systolic pressure; PVR, pulmonary vascular resistance; VTE, venous thromboembolism; WU, Wood unit. (DOCX) [file pmed.1003361.s010.docx]

## **S5 Table. Prevalence of left heart dysfunction, pulmonary dysfunction and VTE, and their association with measures pulmonary vascular dysfunction using common clinical reference limits to define abnormal PASP (>40 mmHg) and PVR (>3.0 WU).**

|  | N abnormal | OR (95%CI) | P value | PAR (95%CI) |
| --- | --- | --- | --- | --- |
| **Abnormal PASP** | Total n=2810 |  |  |  |
| LHD | 798 (28%) | 4.53 (2.71-7.58) | < 0.001 | 18.6 (12.0-24.7) |
| Pulmonary dysfunction | 994 (35%) | 2.37 (1.42-3.97) | 0.001 | 13.6 (4.6-21.8) |
| Prior VTE | 76 (3%) | 1.96 (0.67-5.79) | 0.22 | 0.9 (-0.8, 2.7) [NS] |
| **Abnormal PVR** | Total n=2798 |  |  |  |
| LHD | 793 (28%) | 2.89 (1.28-6.51) | 0.01 | 36.8 (1.62, 59.4) |
| Pulmonary dysfunction | 988 (35%) | 3.35 (1.32-8.51) | 0.01 | 52.9 (5.64, 76.5) |
| Prior VTE | 76 (3%) | 1.24 (0.15-10.0) | 0.84 | 0.7 (-6.97, 7.84) [NS] |

Legend: LHD, left heart disease; VTE, venous thromboembolism; PAR, population attributable risk; NS, not significant. Odds values and p-values are derived from multivariable logistic models containing age, sex, race, visit center LHD, pulmonary dysfunction, and prior VTE.
