# Supplementary material for: Pulmonary vascular dysfunction among people aged over 65 years in the community in the Atherosclerosis Risk In Communities (ARIC) Study: A cross-sectional analysis
Source: PLoS Med. 2020 Oct 15;17(10):e1003361. doi: 10.1371/journal.pmed.1003361 (PMC7561082; doi:10.1371/journal.pmed.1003361)
Supplement: S6 Table — Odds values and p-values are derived from multivariable logistic models containing age, sex, race, visit center, LHD, pulmonary dysfunction, and prior VTE. ESC HFA, European Society of Cardiology Heart Failure Association; HFpEF, incident HF with preserved LVEF; LHD, left heart disease; LVEF, left ventricular ejection fraction; NS, not significant; PAC, pulmonary arterial compliance; PAR, population attributable risk; PASP, pulmonary artery systolic pressure; PVR, pulmonary vascular resistance; VTE, venous thromboembolism. (DOCX) [file pmed.1003361.s011.docx]

## **S6 Table. Prevalence of left heart dysfunction (LHD), pulmonary dysfunction, and venous thromboembolism (VTE), and their association with abnormal PASP, PVR, or PAC after further excluding an additional 227 participants with moderate or severe dyspnea and ESC HFA criterial for HFpEF and those with LVEF < 50%.**

|  | N abnormal | OR (95%CI) | P value | PAR (95%CI) |
| --- | --- | --- | --- | --- |
| **Abnormal PASP** | Total n=2583 |  |  |  |
| LHD | 665 (26%) | 2.14 (1.66-2.76) | <0.001 | 17.9 (11.4-24.0) |
| Pulmonary dysfunction | 865 (33%) | 1.45 (1.14-1.85) | 0.003 | 13.1 (4.1-21.3) |
| Prior VTE | 64 (2%) | 1.52 (0.79-2.95) | 0.21 | 1.0 (-0.8, 2.8) [NS] |
| **Abnormal PVR** | Total n=2573 |  |  |  |
| LHD | 661 (26%) | 1.00 (0.73-1.37) | 0.9 | -0.2 (-8.1, 7.3) [NS] |
| Pulmonary dysfunction | 861 (33%) | 1.31 (0.98-1.74) | 0.06 | 9.9 (-1.3, 19.9) [NS] |
| Prior VTE | 64 (2%) | 1.45 (0.65-3.20) | 0.36 | 0.9 (-1.3, 3.1) [NS] |
| **Abnormal PAC** | Total n=1973 |  |  |  |
| LHD | 531 (27%) | 1.30 (0.93-1.82) | 0.13 | 6.3 (-2.4, 14.3) [NS] |
| Pulmonary dysfunction | 665 (34%) | 1.56 (1.14-2.13) | 0.006 | 17.0 (4.1-28.1) |
| Prior VTE | 53 (3%) | 0.60 (0.21-1.72) | 0.34 | -1.2 (-3.2, 0.8) [NS] |

Legend: LHD, left heart disease; VTE, venous thromboembolism; PAR, population attributable risk; NS, not significant

Odds values and p-values are derived from multivariable logistic models containing age, sex, race, visit center LHD, pulmonary dysfunction, and prior VTE.
