# Supplementary material for: Pulmonary vascular dysfunction among people aged over 65 years in the community in the Atherosclerosis Risk In Communities (ARIC) Study: A cross-sectional analysis
Source: PLoS Med. 2020 Oct 15;17(10):e1003361. doi: 10.1371/journal.pmed.1003361 (PMC7561082; doi:10.1371/journal.pmed.1003361)
Supplement: S7 Table — p-Values were derived from multivariable Cox regression analysis. Model 1 adjusts for age, sex, race, and visit center. Model 2 adjusts for LVEF, LAVi, LVMi, and septal E/e’ in addition to model 1. Model 3 adjusts for hypertension, diabetes, and body mass index in addition to model 2. Model 4 adjusts for PASP in addition to Model 3. HF, heart failure; LAVi, left atrial volume index; LVEF, left ventricular ejection fraction; LVMi, left ventricular mass index; PASP, pulmonary artery systolic pressure. (DOCX) [file pmed.1003361.s012.docx]

**S7 Table. Association of pulmonary hemodynamic measures with incident HF or death post-Visit 5.**

|  | Normal | | | Abnormal | | | Dichotomous | | Continuous | |
| --- | --- | --- | --- | --- | --- | --- | --- | --- | --- | --- |
|  |  |  |  |  |  |  |  |  | (per 1SD increase) | |
|  | N | Events | Event Rate | N | Events | Event Rate | HR | P-value | HR | P-value |
|  |  |  | per 100-person years [95% CI] |  |  | per 100-person years [95% CI] | [95% CI] |  | [95% CI] |  |
| **PASP (mmHg)** |  |  |  |  |  |  |  |  |  |  |
| Unadjusted | 2297 | 313 | 2.55 [2.28-2.85] | 513 | 126 | 4.88 [2.69-3.24] | 1.94 [1.58-2.39] | < 0.001 | 1.37 [1.26-1.49] | < 0.001 |
| Model 1 |  |  |  |  |  |  | 1.68 [1.36-2.07] | < 0.001 | 1.30 [1.19-1.42] | < 0.001 |
| Model 2 |  |  |  |  |  |  | 1.50 [1.20-1.87] | < 0.001 | 1.23 [1.12-1.35] | < 0.001 |
| Model 3 |  |  |  |  |  |  | 1.55 [1.24-1.93] | < 0.001 | 1.25 [1.13-1.37] | < 0.001 |
| **PVR (WU)** |  |  |  |  |  |  |  |  |  |  |
| Unadjusted | 2454 | 365 | 2.78 [2.851-3.08] | 344 | 71 | 4.23 [3.35-5.34] | 1.55 [1.20-2.00] | < 0.001 | 1.21 [1.11-1.32] | < 0.001 |
| Model 1 |  |  |  |  |  |  | 1.38 [1.06-1.79] | 0.016 | 1.15 [1.05-1.26] | 0.002 |
| Model 2 |  |  |  |  |  |  | 1.29 [0.99-1.68] | 0.06 | 1.10 [1.01-1.21] | 0.033 |
| Model 3 |  |  |  |  |  |  | 1.26 [0.96-1.64] | 0.1 | 1.10 [1.00-1.21] | 0.042 |
| Model 4 |  |  |  |  |  |  | 1.08 [0.81-1.42] | 0.6 | 1.02 [0.92-1.13] | 0.7 |
| **PAC (mL/mmHg)** |  |  |  |  |  |  |  |  |  |  |
| Unadjusted | 2044 | 296 | 2.73 [2.43-3.05] | 354 | 110 | 6.58 [5.47-7.94] | 2.51 [1.96-3.21] | < 0.001 | 1.35 [1.19-1.53] | < 0.001 |
| Model 1 |  |  |  |  |  |  | 2.01 [1.56-2.59] | < 0.001 | 1.31 [1.16-1.48] | < 0.001 |
| Model 2 |  |  |  |  |  |  | 1.81 [1.39-2.36] | < 0.001 | 1.24 [1.10-1.40] | < 0.001 |
| Model 3 |  |  |  |  |  |  | 1.80 [1.38-2.35] | < 0.001 | 1.22 [1.08-1.38] | 0.001 |
| Model 4 |  |  |  |  |  |  | 1.48 [1.10-1.98] | 0.009 | 1.09 [0.96-1.25] | 0.19 |

P-values were derived from multivariable Cox regression analysis.

Model 1adjusts for age, sex, race and visit center.

Model 2 adjusts for LVEF, LAVi, LVMI and septal E/e’ in addition to Model 1.

Model 3 adjusts for hypertension, diabetes, and body mass index in addition to Model 2.

Model 4 adjusts for PASP in addition to Model 3.
