# Supplementary material for: Pulmonary vascular dysfunction among people aged over 65 years in the community in the Atherosclerosis Risk In Communities (ARIC) Study: A cross-sectional analysis
Source: PLoS Med. 2020 Oct 15;17(10):e1003361. doi: 10.1371/journal.pmed.1003361 (PMC7561082; doi:10.1371/journal.pmed.1003361)
Supplement: S8 Table — p-Values were derived from multivariable Cox regression models. Model 1 adjusts for age, sex, race, and visit center. Model 2 adjusts for LVEF, LAVi, LVMi, and septal E/e’ in addition to model 1. Model 3 adjusts for hypertension, diabetes, and body mass index in addition to model 2. HFrEF, heart failure with reduced ejection fraction (LVEF < 50%); HFpEF, heart failure with preserved ejection fraction (LVEF ≥ 50%); LAVi, left atrial volume index; LVEF, left ventricular ejection fraction; LVMi, left ventricular mass index. (DOCX) [file pmed.1003361.s013.docx]

## **S8 Table. Continuous association of pulmonary hemodynamic measures with incident HFrEF, HFpEF, and the composite of each of these with death (per 1SD change).**

|  |  |  | Model 1 | |  | Model 2 | |  | Model 3 | |
| --- | --- | --- | --- | --- | --- | --- | --- | --- | --- | --- |
|  | Total n | Number of events | HR [95% CI] | p-value |  | HR [95% CI] | p-value |  | HR [95% CI] | p-value |
| **PASP (mmHg)** |  |  |  |  |  |  |  |  |  |  |
| HFrEF | 2810 | 62 | 0.94 [0.71-1.23] | 0.65 |  | 0.93 [0.71-1.23] | 0.62 |  | 0.90 [0.68-1.19] | 0.46 |
| HFpEF |  | 68 | 1.85 [1.54-2.22] | < 0.001 |  | 1.56 [1.26-1.93] | < 0.001 |  | 1.57 [1.27-1.96] | < 0.001 |
| HFrEF or death |  | 378 | 1.23 [1.11-1.35] | < 0.001 |  | 1.18 [1.06-1.31] | 0.002 |  | 1.20 [1.08-1.34] | 0.001 |
| HFpEF or death |  | 387 | 1.32 [1.21-1.45] | < 0.001 |  | 1.25 [1.14-1.38] | < 0.001 |  | 1.28 [1.15-1.41] | < 0.001 |
| **PVR (WU)** |  |  |  |  |  |  |  |  |  |  |
| HFrEF | 2729 | 62 | 1.20 [0.95-1.52] | 0.12 |  | 0.97 [0.77-1.21] | 0.78 |  | 0.97 [0.77-1.21] | 0.76 |
| HFpEF |  | 66 | 1.30 [1.05-1.62] | 0.016 |  | 1.24 [0.99-1.55] | 0.06 |  | 1.23 [0.98-1.53] | 0.07 |
| HFrEF or death |  | 375 | 1.16 [1.05-1.28] | 0.003 |  | 1.11 [1.00-1.22] | 0.046 |  | 1.10 [1.00-1.22] | 0.06 |
| HFpEF or death |  | 384 | 1.15 [1.05-1.27] | 0.004 |  | 1.12 [1.02-1.24] | 0.018 |  | 1.12 [1.01-1.24] | 0.025 |
| **PAC (mL/mmHg)** |  |  |  |  |  |  |  |  |  |  |
| HFrEF | 2044 | 41 | 1.69 [1.16-2.46] | 0.007 |  | 1.34 [0.92-1.93] | 0.13 |  | 1.32 [0.92-1.89] | 0.14 |
| HFpEF |  | 51 | 1.16 [0.86-1.57] | 0.33 |  | 1.07 [0.81-1.42] | 0.64 |  | 1.09 [0.82-1.44] | 0.55 |
| HFrEF or death |  | 283 | 1.39 [1.22-1.60] | < 0.001 |  | 1.31 [1.15-1.50] | < 0.001 |  | 1.28 [1.12-1.47] | < 0.001 |
| HFpEF or death |  | 294 | 1.31 [1.15-1.49] | < 0.001 |  | 1.26 [1.10-1.43] | 0.001 |  | 1.23 [1.08-1.40] | 0.002 |

Legend: HFrEF, heart failure with reduced ejection fraction (LVEF <50%); HFpEF, heart failure with preserved ejection fraction (LVEF ≥50%). P-values were derived from multivariable Cox regression models.

Model 1 adjusts for age, sex, race and visit center.

Model 2 adjusts for LVEF, LAVi, LVMI and septal E/e’ in addition to Model 1.

Model 3 adjusts for hypertension, diabetes, and body mass index in addition to Model 2.
