# Supplementary material for: Pulmonary vascular dysfunction among people aged over 65 years in the community in the Atherosclerosis Risk In Communities (ARIC) Study: A cross-sectional analysis
Source: PLoS Med. 2020 Oct 15;17(10):e1003361. doi: 10.1371/journal.pmed.1003361 (PMC7561082; doi:10.1371/journal.pmed.1003361)
Supplement: S9 Table — p-Values were derived from inverse probability weighted multivariable Cox regression analysis. Model 1 adjusts for age, sex, race, and visit center. Model 2 adjusts for LVEF, LAVi, LVMi, and septal E/e’ in addition to model 1. Model 3 adjusts for hypertension, diabetes, and body mass index in addition to model 2. HF, heart failure; LAVi, left atrial volume index; LVEF, left ventricular ejection fraction; LVMi, left ventricular mass index. (DOCX) [file pmed.1003361.s014.docx]

## **S9 Table. Association of pulmonary hemodynamic measures with incident HF or death post-Visit 5 using inverse probability weights.**

|  | Normal | | | Abnormal | | | Dichotomous | | Continuous | |
| --- | --- | --- | --- | --- | --- | --- | --- | --- | --- | --- |
|  |  |  |  |  |  |  |  |  | (per 1SD increase) | |
|  | N | Events | Event Rate | N | Events | Event Rate | HR | P-value | HR | P-value |
|  |  |  | per 100-person years [95% CI] |  |  | per 100-person years [95% CI) | [95% CI) |  | [95% CI) |  |
| **PASP (mmHg)** |  |  |  |  |  |  |  |  |  |  |
| Unadjusted | - | - | 2.86 [2.56-3.21] | - | - | 5.38 [4.53-6.43] | 1.91 [1.54-2.36] | < 0.001 | 1.37 [1.24-1.50] | < 0.001 |
| Model 1 |  |  |  |  |  |  | 1.62 [1.30-2.02] | < 0.001 | 1.29 [1.17-1.42] | < 0.001 |
| Model 2 |  |  |  |  |  |  | 1.46 [1.16-1.84] | 0.001 | 1.23 [1.10-1.38] | < 0.001 |
| Model 3 |  |  |  |  |  |  | 1.51 [1.20-1.92] | 0.001 | 1.25 [1.12-1.40] | < 0.001 |
| **PVR (WU)** |  |  |  |  |  |  |  |  |  |  |
| Unadjusted | - | - | 3.12 [2.81-3.46] | - | - | 4.75 [3.80-6.02] | 1.55 [1.20-2.01] | 0.001 | 1.24 [1.13-1.35] | < 0.001 |
| Model 1 |  |  |  |  |  |  | 1.38 [1.06-1.81] | 0.019 | 1.17 [1.07-1.28] | < 0.001 |
| Model 2 |  |  |  |  |  |  | 1.30 [0.99-1.72] | 0.06 | 1.13 [1.03-1.23] | 0.009 |
| Model 3 |  |  |  |  |  |  | 1.27 [0.96-1.68] | 0.1 | 1.13 [1.03-1.24] | 0.012 |
| **PAC (mL/mmHg)** |  |  |  |  |  |  |  |  |  |  |
| Unadjusted | - | - | 2.72 [2.40-3.09] | - | - | 7.02 [5.71-8.71] | 2.65 [2.06-3.42] | < 0.001 | 1.40 [1.20-1.64] | < 0.001 |
| Model 1 |  |  |  |  |  |  | 2.11 [1.61-2.77] | < 0.001 | 1.34 [1.16-1.55] | < 0.001 |
| Model 2 |  |  |  |  |  |  | 1.93 [1.45-2.59] | < 0.001 | 1.28 [1.11-1.47] | 0.001 |
| Model 3 |  |  |  |  |  |  | 1.92 [1.44-2.58] | < 0.001 | 1.25 [1.08-1.45] | 0.002 |

P-values were derived from inverse probability weighted multivariable Cox regression analysis.

Model 1adjusts for age, sex, race and visit center.

Model 2 adjusts for LVEF, LAVi, LVMi and septal E/e’ in addition to Model 1.

Model 3 adjusts for hypertension, diabetes, and body mass index in addition to Model 2.
