# Supplementary material for: Pulmonary vascular dysfunction among people aged over 65 years in the community in the Atherosclerosis Risk In Communities (ARIC) Study: A cross-sectional analysis
Source: PLoS Med. 2020 Oct 15;17(10):e1003361. doi: 10.1371/journal.pmed.1003361 (PMC7561082; doi:10.1371/journal.pmed.1003361)
Supplement: S10 Table — p-Values were derived from multivariable Cox regression model adjusted for age, sex, race, visit center, BMI, hypertension, diabetes, LVEF, LAVi, LVMi, and septal E/e’. BMI, body mass index; ESC HFA, European Society of Cardiology Heart Failure Association; HF, heart failure; HFrEF, heart failure with reduced ejection fraction (LVEF < 50%); HFpEF, heart failure with preserved ejection fraction (LVEF ≥ 50%); LAVi, left atrial volume index; LVEF, left ventricular ejection fraction; LVMi, left ventricular mass index; PAC, pulmonary arterial compliance; PASP, pulmonary artery systolic pressure; PVR, pulmonary vascular resistance. (DOCX) [file pmed.1003361.s015.docx]

## **S10 Table. Association of abnormalities of pulmonary vascular measures (PASP, PVR, PAC) with incident HF overall, incident HFpEF, incident HFrEF, or the composite of death with each of these when further excluding an additional 227 participants with moderate or severe dyspnea and ESC HFA criterial for HFpEF and those with LVEF <50%.**

| Abnormal pulmonary vasculature measures | Clinical outcome | Number of events  Normal/Abnormal | Event rate (per 100 person years)  Normal/Abnormal | HR [95% CI] |
| --- | --- | --- | --- | --- |
| Abnormal PASP Total n=2583 | HF | 74 / 36 | 0.64 / 1.62 | 1.90 [1.24-2.92] |
|  | HFrEF | 32 / 8 | 0.28 / 0.36 | 1.12 [0.49-2.56] |
|  | HFpEF | 31 / 19 | 0.27 / 0.85 | 2.24 [1.21-4.14] |
|  | HF or death | 266 / 88 | 2.29 / 3.95 | 1.44 [1.11-1.86] |
|  | HFrEF or death | 237 / 68 | 2.04 / 3.05 | 1.29 [0.96-1.72] |
|  | HFpEF or death | 241 / 76 | 2.08 / 3.41 | 1.35 [1.02-1.78] |
|  |  |  |  |  |
| Abnormal PVR Total n=2573 | HF | 94 / 16 | 0.77 / 1.06 | 1.14 [0.66 -1.99] |
|  | HFrEF | 35 / 5 | 0.29 / 0.32 | 0.74 [0.28-1.95] |
|  | HFpEF | 43 / 7 | 0.35 / 0.46 | 1.20 [0.52-2.77] |
|  | HF or death | 294 / 59 | 2.40 / 3.89 | 1.41 [1.05-1.90] |
|  | HFrEF or death | 252/ 52 | 2.06 / 3.43 | 1.43 [1.04-1.96] |
|  | HFpEF or death | 263 / 53 | 2.15 / 3.49 | 1.43 [1.04-1.96] |
|  |  |  |  |  |
| Abnormal PAC Total n=1973 | HF | 61 / 20 | 0.65/ 1.65 | 2.00 [1.18-3.40] |
|  | HFrEF | 17 / 9 | 0.18 / 0.74 | 2.60 [1.11-6.06] |
|  | HFpEF | 31 / 7 | 0.33 / 0.58 | 1.53 [0.64-3.66] |
|  | HF or death | 212 / 56 | 2.27 / 4.59 | 1.53 [1.12-2.09] |
|  | HFrEF or death | 182 / 47 | 1.95 / 3.85 | 1.44 [ 1.02-2.03] |
|  | HFpEF or death | 194 / 46 | 2.07 / 3.77 | 1.34 [0.95-1.90] |

Legend: HF, heart failure; HFrEF, heart failure with reduced ejection fraction (LVEF <50%); HFpEF, heart failure with preserved ejection fraction (LVEF ≥50%). Hazard ratio and 95% confidence intervals were derived from multivariable Cox regression model adjusted for age, sex, race, visit center, BMI, hypertension, diabetes, LVEF, LAVi, LVMi and septal E/e’.
