# Supplementary material for: Pulmonary vascular dysfunction among people aged over 65 years in the community in the Atherosclerosis Risk In Communities (ARIC) Study: A cross-sectional analysis
Source: PLoS Med. 2020 Oct 15;17(10):e1003361. doi: 10.1371/journal.pmed.1003361 (PMC7561082; doi:10.1371/journal.pmed.1003361)
Supplement: S11 Table — p-Values were derived from multivariable Cox regression model adjusted for age, sex, race, visit center, BMI, hypertension, diabetes, LVEF, LAVi, LVMi, and septal E/e’. BMI, body mass index; HF, heart failure; HFrEF, heart failure with reduced ejection fraction (LVEF < 50%); HFpEF, heart failure with preserved ejection fraction (LVEF ≥ 50%); LAVi, left atrial volume index; LVEF, left ventricular ejection fraction; LVMi, left ventricular mass index. (DOCX) [file pmed.1003361.s016.docx]

## **S11 Table. Association of pulmonary hemodynamic measures based on established reference limits with incident HF or death post-Visit 5.**

| Abnormal pulmonary vasculature measures | Clinical outcome | Number of events  Normal/Abnormal | Event rate (per 100 person-years)  Normal/Abnormal | HR [95% CI] |
| --- | --- | --- | --- | --- |
| Total n=2810  Abnormal PASP 86 (3.1%) | HF | 142 / 12 | 0.98 / 3.14 | 2.20 [1.15-4.22] |
|  | HFrEF | 60 / 2 | 0.42 / 0.52 | 0.90 [0.21-3.88] |
|  | HFpEF | 61 / 7 | 0.42 / 1.83 | 2.39 [0.96-5.98] |
|  | HF or death | 407 / 32 | 2.81 / 8.35 | 2.55 [1.72-3.77] |
|  | HFrEF or death | 351 / 27 | 2.42 / 7.04 | 2.65 [1.73-4.06] |
|  | HFpEF or death | 357 / 30 | 2.46 / 7.83 | 2.74 [1.82-4.12] |
|  |  |  |  |  |
| Total n=2798  Abnormal PVR 30 (1.1%) | HF | 146 / 6 | 1.00 / 4.34 | 1.63 [0.66 -4.02] |
|  | HFrEF | 60 / 2 | 0.41 / 1.45 | 0.90 [0.19-4.25] |
|  | HFpEF | 64 / 2 | 0.43 / 1.45 | 1.10 [0.19-6.34] |
|  | HF or death | 420 / 10 | 2.90 / 7.23 | 1.58 [0.82-3.04] |
|  | HFrEF or death | 367/ 8 | 2.50 / 5.79 | 1.56 [0.76-3.22] |
|  | HFpEF or death | 376 / 8 | 2.56 / 5.79 | 1.55 [0.75-3.21] |

Legend: HF, heart failure; HFrEF, heart failure with reduced ejection fraction (LVEF <50%); HFpEF, heart failure with preserved ejection fraction (LVEF ≥50%). P-values were derived from multivariable Cox regression model adjusted for age, sex, race, visit center, BMI, hypertension, diabetes, LVEF, LAVi, LVMi and septal E/e’.
