# Supplementary material for: Pulmonary vascular dysfunction among people aged over 65 years in the community in the Atherosclerosis Risk In Communities (ARIC) Study: A cross-sectional analysis
Source: PLoS Med. 2020 Oct 15;17(10):e1003361. doi: 10.1371/journal.pmed.1003361 (PMC7561082; doi:10.1371/journal.pmed.1003361)
Supplement: S1 Text — (DOCX) [file pmed.1003361.s017.docx]

**S1 Text**

*Inverse probability of attrition weighting for sensitivity analysis*

To account for potential bias due to selective attrition related to either unmeasurable TR jet velocity on Visit 5 echocardiography or Visit 5 non-attendance, we performed a sensitivity analysis using inverse probability of attrition weighting. Visit 5 non-attendance was modeled among participants alive at the initiation of Visit 5 using the following covariates from Visit 1: age, gender, race, study center, systolic and diastolic blood pressure, heart rate, body mass index, smoking and drinking status, diabetes, hypertension, and chronic kidney disease. The resulting calculated weights were incorporated into multivariable models for prevalence estimates of pulmonary vascular dysfunction, the association of measures of cardiac structure and function with each pulmonary vascular measure, and the relationship of each pulmonary vascular measure with incident HF or death.

*Ascertainment of LVEF*

LVEF from the time of hospitalization was ascertained by trained chart abstractors as the lowest assessed LVEF at the time of the HF admission, preferably by transthoracic echocardiography. When no LVEF assessment was performed or available from the heart failure admission, the most recent and lowest LVEF assessed within the prior to 6 months was used if there was no intercurrent myocardial infarction. The HF hospital record abstraction form can be found at the ARIC website.

*Definition of HFpEF for sensitivity analysis*

To further assess the potential impact of including persons with undiagnosed HF, we performed an additional sensitivity analysis further excluding participants who self-reported presence of moderate to severe dyspnea at Visit 5 and who met HFpEF diagnostic criteria of the European Society of Cardiology (ESC) Heart Failure Association (HFA) based on a score of ≥5. Dyspnea was assessed at Visit 5 using the modified Medical Research Council scale (mMRC) as previously described [^[[1]](#endnote-1)^,^[[2]](#endnote-2)^], on a scale of 0 to 4. Zero indicated a positive answer to “Are you troubled by shortness of breath except on strenuous exertion?”; 1, a positive answer to “Are you short of breath when hurrying on the level or walking up a slight hill?”; 2, a positive answer to “Do you have to walk slower than most people on the level because of breathlessness? Do you have to stop after a mile or so (or after half an hour) on the level at your own pace?”; 3, a positive answer to “Do you have to stop for breath after walking about 100 yards (or after a few minutes) on the level?”; and 4, a positive answer to “Are you too breathless to leave the house, or breathless after undressing?” Moderate dyspnea was defined as an mMRC score of 2 or 3, and severe dyspnea as an mMRC score of 4 [^[[3]](#endnote-3)^,^[[4]](#endnote-4)^,^[[5]](#endnote-5)^]. ESC HFA score for HFpEF specifies major and minor criteria based on echocardiographic functional measures (including septal and lateral e’, TR velocity, E/e’, longitudinal strain), echocardiographic morphologic features (including LAVi, LVMi, RWT, and LV wall thickness), and biomarker levels (NT-proBNP, BNP) [^[[6]](#endnote-6)^]. Each major criteria is assigned 2 points and each minor criteria is assigned 1 point. A score of ≥5 is considered consistent with HFpEF.

Reference

1. Fletcher CM, Elmes PC, Fairbairn AS, Wood CH. The significance of respiratory symptoms and the diagnosis of chronic bronchitis in a working population. BMJ. 1959;2(5147):257-266. [↑](#endnote-ref-1)
2. Ramalho SHR, Santos M, Claggett B, Matsushita K, Kitzman DW, Loehr L, Solomon SD, Skali H, Shah AM. Association of Undifferentiated Dyspnea in Late Life With Cardiovascular and Noncardiovascular Dysfunction: A Cross-sectional Analysis From the ARIC Study. JAMA Netw Open 2019;2:e195321. [↑](#endnote-ref-2)
3. MinerB,TinettiME,VanNessPH,etal.Dyspneaincommunity-dwellingolderpersons:amultifactorialgeriatric health condition. J Am Geriatr Soc. 2016;64(10):2042-2050. [↑](#endnote-ref-3)
4. Hegendörfer E, Vaes B, Matheï C, Van Pottelbergh G, Degryse JM. Correlates of dyspnoea and its association with adverse outcomes in a cohort of adults aged 80 and over. Age Ageing. 2017;46(6):994-1000. [↑](#endnote-ref-4)
5. OelsnerEC,LimaJA,KawutSM,etalNoninvasivetestsforthediagnosticevaluationofdyspneaamong outpatients: the Multi-Ethnic Study of Atherosclerosis lung study. Am J Med. 2015;128(2):171-180.e5. [↑](#endnote-ref-5)
6. Pieske B, Tschöpe C, de Boer RA, Fraser AG, Anker SD, Donal E, Edelmann F, Fu M, Guazzi M, Lam CSP, Lancellotti P, Melenovsky V, Morris DA, Nagel E, Pieske-Kraigher E, Ponikowski P, Solomon SD, Vasan RS, Rutten FH, Voors AA, Ruschitzka F, Paulus WJ, Seferovic P, Filippatos G. How to diagnose heart failure with preserved ejection fraction: the HFA-PEFF diagnostic algorithm: a consensus recommendation from the Heart Failure Association (HFA) of the European Society of Cardiology (ESC). Eur Heart J. 2019 Oct 21;40(40):3297-3317. [↑](#endnote-ref-6)
