# Supplementary material for: Pulmonary vascular dysfunction among people aged over 65 years in the community in the Atherosclerosis Risk In Communities (ARIC) Study: A cross-sectional analysis
Source: PLoS Med. 2020 Oct 15;17(10):e1003361. doi: 10.1371/journal.pmed.1003361 (PMC7561082; doi:10.1371/journal.pmed.1003361)
Supplement: Attachment — Submitted filename: Response to Reviewers_PMEDICINE-D-19-04388.docx [file pmed.1003361.s018.docx]

**Response to Reviewers**

We appreciate the valuable comments and suggestions of the Editor and Reviewers. This letter details the changes to our manuscript that we have made in response to each of the comments and suggestions provided.

**Request from the editors;**

**1. Please revise your title according to PLOS Medicine's style, placing the study design in the subtitle (ie, after a colon). We suggest “Pulmonary vascular dysfunction among people aged over 65 years in the community (ARIC): a cross-sectional analysis” or similar. NB) Age of over 65 has been taken from line 81 - please correct age based on the lowest age among all participants included in this study.**

We have revised our title as, ‘Pulmonary vascular dysfunction among people aged over 65 years in the community in the Atherosclerosis Risk In Communities (ARIC) Study: a cross-sectional analysis’ as requested.

**2. Abstract Background:**

a. Please expand upon the context of why the study is important.

We have now added the following sentence to the abstract to expand on the context of why the study is important:

Line 26-27: “Heart failure (HF) risk is highest in late-life, and impaired pulmonary vascular function is a risk factor for HF.”

b. Line 30 - please define LVEF. Please clarify what is meant by (LVEF ≥ or <50%)

We have now clearly defined LVEF.

Line 32: “left ventricular ejection fraction [LVEF]”

**2. Abstract Methods and Findings:**

a. Please include further details of the study setting and main/secondary outcome measures. We have added additional details as requested to the abstract.

Line 36-45:

“This is an ongoing, observational study which recruited 15,792 persons aged 45 to 64 years between 1987 and 1989 (Visit 1) from four representative communities in the United States: Minneapolis, MN; Jackson, MS; Hagerstown, MD; Forsyth County, NC. This current analysis included 2,810 individuals aged 66 to 90 years old, free of HF who underwent echocardiography at the 5^th^ study visit (June 8^th^, 2011- August 28^th^, 2013) and had measurable tricuspid regurgitation by spectral Doppler. Echocardiography-derived pulmonary artery systolic pressure (PASP), pulmonary vascular resistance (PVR), and pulmonary arterial compliance (PAC) were measured. The main outcome was incident HF after Visit 5, and secondary endpoints were incident HFpEF and HFrEF.”

b. Please quantify the main results (with 95% CIs and p values).

We have now added effect estimates with 95% CI and p values throughout the abstract. (See lines 52-58).

c. Line 34/35 - please omit one instance of the word ‘prospective’ to avoid repetition

We have now removed the word ‘prospective’ as requested. (See line 36)

d. Line 35 - please clarify what communities

We now specify the four communities from which participants were recruited. Please refer to the response to Editor comment 2.a. above and Line 38 in the abstract.

e. Line 37 - please define ‘elderly’ by specifying the age of participants

We now specify the age of the study participants. Please refer to the response to Editor comment 2.a. above and Line 40 in the abstract.

f. Line 38 - please provide dates between which data was collected for this study

We now specify the dates between which data was collected for this study. Please refer to the response to Editor comment 2.a. above and Line 40-41 in the abstract.

g. Line 40 - please define fully PVR and PAC

We now fully define PVR and PAC. Please refer to the response to Editor comment 2.a. above and Line 42-43 in the abstract.

h. Line 41 - please define what 76±5 years represents e.g. mean ± SD

We have now defined what 75±5 years represents, as follows:

Line 45: “The mean±SD age was 76±5 years”

i. Line 42 - please define WU

We have now defined WU (see Line 47).

j. Line 49 - please define HFpEF and HFrEF

We now define HFpEF and HFrEF. Please refer to the response to Editor comment 2.a. above and Line 44-45 in the abstract.

k. In the last sentence of the Abstract Methods and Findings section, please describe the main limitation(s) of the study's methodology.

We have now added the following sentence to the end of the Abstract Methods and Findings section to describe the study’s main limitations:

Line 60-64: “Major limitations include the use of echo Doppler to estimate pulmonary hemodynamic measures, inclusions bias related to detectable tricuspid regurgitation, and survivor bias related to the cohort age.”

**3. Abstract Conclusions**

Please begin your Abstract Conclusions with "In this study, we observed ..." or similar. Please address the study implications, emphasizing what is new without overstating your conclusions.

a. We have modified the conclusions as follows:

“In this study, we observed abnormalities of PASP, PVR, and PAC in 12-18% of elders in the community. Higher PASP and lower PAC were independently predictive of incident HF. Abnormally high PASP predicted incident HFpEF but not HFrEF, while abnormal PAC predicted HFrEF.” (Line 65-68)

b. Line 55/336 - ‘precede’ would be more conventional.

We have changed the word ‘antecede’ to ‘precede’ as suggested. (Line 69, 106, 412, 514)

c. At this stage, we ask that you include a short, non-technical Author Summary of your research to make findings accessible to a wide audience that includes both scientists and non-scientists. Please include a final bullet point under ‘What do these findings mean?’ to describe the main limitations(s) of the study.

We have now added a non-technical Author Summary immediately following Abstract in the revised manuscript.

Lines 72-113:

“Why was this study done?

- Abnormal pressure or function of the blood vessels in the lungs is associated with risk of developing heart failure. These blood vessels in the lungs can be characterized by pressure, resistance, and compliance.
- Alterations in heart and lung function can influence the pulmonary blood vessels.
- The risk of heart failure is highest in late life. However, little is known about the association of heart and lung dysfunction with pulmonary vascular measures, and the association of pulmonary vascular measures with heart failure risk, among elders in the community.

What did the researchers do and find?

- We performed a cross-sectional and time to event analysis in a large community-based cohort with participants aged 66 to 90 years old.
- We measured the cross-sectional association of left heart disease, pulmonary dysfunction, and venous thromboembolism with abnormalities in these three major pulmonary vasculature measures (i.e. pulmonary pressure, resistance, and compliance)
- The presence of left heart disease was associated with abnormally higher pulmonary pressure. Pulmonary dysfunction was associated with abnormalities in all three pulmonary vasculature measures (i.e. pressure, resistance, and compliance).
- We performed time-to-event analysis to investigate the association of abnormalities in pulmonary vasculature measures with future development of heart failure and heart failure subtypes (heart failure with preserved or reduced left ventricular ejection fraction).
- We found that higher pulmonary pressure and lower compliance were associated with risk of heart failure. Higher pulmonary pressure was associated with greater risk of heart failure with preserved ejection fraction but not with reduced ejection fraction. Lower pulmonary arterial compliance was more strongly associated with heart failure with reduced ejection fraction.

What do these findings mean?

- These findings suggest that abnormalities of pulmonary pressure and compliance may precede the development of heart failure, and are associated with increased risk for heart failure.
- Further research is needed to better understand whether, and how, these abnormalities in the pulmonary vasculature directly contribute to heart failure development, and whether this differs by heart failure subtype.
- The main limitations of this study include potential error in pulmonary vascular measures due to the methodology used, and the selection of participants in this analysis which may limit the generalizability of these findings.”

**4. Introduction**

a. Please expand upon the need for and potential importance of your study. Indicate whether your study is novel and how you determined that. If there has been a systematic review of the evidence related to your study (or you have conducted one), please refer to and reference that review and indicate whether it supports the need for your study.

To our knowledge, no systematic review has investigated the relationships between pulmonary artery pressure, resistance, and compliance with risk of incident heart failure and heart failure phenotype. A number of studies have described the association of higher PASP with incident HF. However, to our knowledge, prior studies have not comprehensively assessed the correlates and prognostic importance of pulmonary pressure, resistance, and compliance for heart failure among persons in late life. We have modified the Introduction as follows to clarify the novelty of our study:

Introduction, line 128-130: “While prior studies have described the association of PASP with incident HF, little data is available regarding the prevalence, causes, and prognostic implications of pulmonary hypertension and vascular dysfunction in the elderly.”

b. Line 63 / 64 - please define PVR, PAC and LV

These abbreviations have now been defined in Line 119, 120, and 121.

c. Line 67 - please define HF

This abbreviation has now defined in Line 123.

**5. Methods**

a. Please ensure that the study is reported according to the STROBE guideline, and include the completed STROBE checklist as Supporting Information. Please add the following statement, or similar, to the Methods: "This study is reported as per the Strengthening the Reporting of Observational Studies in Epidemiology (STROBE) guideline (S1 Checklist)."

We have added the requested sentence to the end of the methods section (Line 266-267). We have added the STROBE checklist to the Supplemental materials.

b. Did your study have a prospective protocol or analysis plan? Please state this (either way) early in the Methods section. If a prospective analysis plan was used in designing the study, please include the relevant prospectively written document with your revised manuscript as a Supporting Information file to be published alongside your study, and cite it in the Methods section. If no such document exists, please make sure that the Methods section transparently describes when analyses were planned, when/why any data-driven changes to analyses took place, and what those changes were.

The overall ARIC study was based on a prospective protocol aiming to investigate the etiology of atherosclerosis, novel risk factors, and clinical sequelae as previously published [^[1]^]. Visit 5 was the first examination to include transthoracic echocardiography at all study Field Centers, and the objectives and design of incorporating echocardiography at this Visit have been previously published [^[2]^]. For the analysis presented in this manuscript, no specific prospective protocol existed. However, a manuscript proposal form was written, submitted, and approved by the ARIC publications committee which included details of data collection and planned analyses prior to conducting the analysis. By the time manuscript analysis began 8/2017, heart failure events post-Visit 5 were available and the analysis plan was altered to include the association of pulmonary vascular measures with incident heart failure events. We have now modified the Methods section to reflect this as follows:

Methods section, Study Population subsection, Line 157-159:

“Prospective protocols for the ARIC study overall and echocardiography at Visit 5 have been previously published. This analysis did not have a specific prospective protocol.”

c. Where interviews were conducted, please provide a copy of the proforma(s) used in your supplementary information.

For our primary exposure and outcomes, interviews were not performed. However, as a part of ARIC visit and for the collection of covariate data, interviews were performed. Detailed interview data collection forms are available at the ARIC website (<https://sites.cscc.unc.edu/aric/desc_pub>). Patient reported outcomes were not included. We have modified the Methods section to reflect this as follows:

Methods section, Clinical Covariates subsection, Line 203-204:

“Detailed interview data collection forms are available at the ARIC website (<https://sites.cscc.unc.edu/aric/desc_pub>).”

d. Line 90 - please provide dates between which data was collected for this study

We now provide the data collection dates as requested.

Line 147: “Between June 2^nd^ 2011 and August 30^th^ 2013,”

**6. Results**

a. Please provide 95% CIs in addition to p values. Line 218 - please provide p values for each of the measures, rather than grouping all as P ≤0.003. Line 251 / 278 / 280 - please provide p values for each of the measures, rather than grouping all as P ≤0.001

We now provide 95% CI and p values throughout the Results section. For all four associations referenced, we now present P values. Please refer to Results section lines 276-278, 281-283, 348-352.

b. Line 277 - Please indicate which factors are adjusted for

We now specify the adjustment covariates as follows:

Results section, Relationship of pulmonary vascular hemodynamics with NT-proBNP, RV function, and incident HF subsection:

Line 346--349: “These associations persisted after adjustment for participant demographics (age, sex, race, visit center and BMI), hypertension, diabetes, and LV measures including LVEF, LVMi, LAVi and E/e”

Line 366: “In models adjusted for age, sex, race, visit center, BMI, hypertension, diabetes, and LV measures (LVEF, LAVi, LVMI and septal E/E’)”

c. Please define the length of follow up (eg, in mean, SD, and range).

All participants were followed from the time of Visit 5 through December 31^st^, 2018. The follow-up time presented was the mean ± standard deviation in years. The range of the follow-up time was 6.6 years and is now clearly stated in line 363. We have also added the following sentence to the methods section:

Line 226-227; “For this analysis, all participants were followed through December 31^st^, 2018.”

d. Lines 282-284 - please clarify whether the 425 participants who died are included in the 547 participants for whom ‘ Death or HF hospitalization occurred’. Please clarify how all numbers presented here relate to each other

Participants with an incident HF event may have subsequently died. For the composite of HF hospitalization or death, the first event was counted. We have now updated the manuscript text to clarify as follows:

Line 363-366: “161 participants developed incident HF (71 HFpEF, 65 HFrEF, 25 HF with unknown LVEF), and 344 died. Incident HF or death occurred in 446 (59 with incident HF prior to death: 24 HFpEF, 26 HFrEF, 9 HF with unknown LVEF).”

e. All tables and figures - Please define all abbreviations used in the legend of each table/figure. When a p value is given, please specify the statistical test used to determine it, in the table/figure legend.

All abbreviations in the tables/figures are now defined in each legend along with statistical test being used to produce p-values. Please see tables and figures in the main manuscript and supplemental materials.

f. All tables - Please present numerators and denominators, in addition to percentages.

We now provided the number of participants along with percentage in all Tables. Please see modified tables and figures in the main manuscript and supplemental materials. We are unable to provide both of these values in Supplemental Table 9 which demonstrates results using inverse probability weights, in which weighted proportions only are provided.

g. Table 1 - Please ensure that it is clear what results including ± represent (e.g. standard deviation)

We have now added a note in the table legend clarifying that all continuous variables are shown with mean ± standard deviation, except for non-normally distributed variables where median with interquartile range in square brackets are presented.

h. Figure 1 - Please incorporate panel C alongside panels A and B into one figure, as Figure 1; or divide into two separate figures entirely

We have now separated former figure 1 panel C from Figure 1 and now label it as Figure 2.

i. Figures 3 and S4 - please avoid using red and green together, to improve accessibility to those with color blindness

The color green has now been changed to orange in Figure 4.

**7. Discussion**

a. Please discuss the implications of your study and next steps for research, clinical practice, and/or public policy.

We have added the following text to the Discussion section to highlight the potential implications of these finding for clinical practice and future research into HF:

Line 470-473: “Comprehensive assessment of pulmonary arterial pressure, resistance, and compliance may ultimately help clinicians better identify older adults at heighted risk for HF, and better target patients for interventions to prevent and treat HF through more detailed phenotyping of the pulmonary vasculature.”

b. Line 391 - please revise sentence beginning ‘This is particularly important...’ to clarify meaning

We have now modified this sentence as follows (line 466-470):

Originally: “This is particularly important given data suggesting that pulmonary vascular disease defines a distinct HFpEF phenotype and the presence of at least 8 ongoing phase 2-3 RCTs of agents targeting PH in HFpEF.”

Revised: “This is particularly important as the results of prior analyses using hierarchical clustering approaches suggest that pulmonary vascular disease identifies a distinct HFpEF phenogroup. Furthermore, at least 8 ongoing phase 2-3 RCTs are testing agents targeting PH in HFpEF.”

c. Please remove subheading for Conclusion

The subheading for Conclusion is now removed. (Line 507)

**Reviewer #1: Statistical review**

1. Abstract: what are the figures following the +/- standard deviations, or standard errors, or something else? This is also applicable to the results.

Values after the ± were the standard deviation. We have now specified this throughout the abstract and Results section. Please refer to manuscript Lines 45-47.

2. Abstract/results - if the abnormality limits were below 10th or above 90th percentiles, then wouldn't 20% of participants be expected to be 'abnormal' even if they were consistent with healthy individuals? This also is relevant to the results on bottom paragraph of page 13 - this actually seems like there is lower abnormality than there should be.

We apologize for the confusion. We have now clarified that ‘abnormal’ was defined as a PASP and PVR above the 90^th^ percentile limit, and PAC below the 10^th^ percentile limit, of the distribution in the low risk subgroup derived using quantile regression. We have made the following changes to the Methods section to clarify:

Methods section, Statistical Methods sub-section, Line 230-235:

Originally:

“Echocardiographic measures of pulmonary hemodynamics were described in the low risk reference subgroup overall and stratified by sex using quantile regression (STATA qreg) to define the 10^th^ and 90^th^ percentile limits with associated 95% confidence limits, which were considered reference limits for the overall ARIC sample.”

Revised:

“Echocardiographic measures of pulmonary hemodynamics were described in the low risk reference subgroup overall and stratified by sex using quantile regression (STATA qreg) to define the 10^th^ and 90^th^ percentile limits with associated 95% confidence limits. Abnormal PASP and PVR were defined as values above the resulting 90^th^ percentile limits, while abnormal PAC was defined as values below the 10^th^ percentile limit.”

3. Abstract - I would recommend summaries of effect sizes of association, plus confidence intervals, between PASP, PVR and PAC and time to incident HF/death are given. For the first time a confidence interval is given (presumably the square bracket ranges are CIs), I'd mention that it is a confidence interval.

Hazard ratios, 95% confidence intervals and p-values have now been added to the abstract as follows:

Line 51-58: “PASP, PVR, and PAC were each predictive of incident HF or death (HR per standard deviation 1.3 [95% CI 1.1-1.7], p <0.001; 1.1 [1.0-1.2], p=0.04; 1.2 [1.1-1.4], p=0.001, respectively) independent of left ventricular (LV) measures. Elevated pulmonary pressure was predictive of incident HFpEF (HFpEF: 2.4 [1.4-4.0, p=0.001]) but not HFrEF (1.4 [0.8-2.5, p=0.31]). Abnormal PAC predicted both (HFpEF: 2.0 [1.0-4.0, p=0.05], HFrEF: 2.8 [1.4-5.5, p=0.003]), while abnormal PVR was not predictive of either (HFpEF: 0.7 [0.3-1.4, p=0.30], HFrEF: 0.9 [0.4-2.0, p=0.85]).”

4. Abstract - the acronyms on line 49 are not defined in the abstract (nor in the paper).

We have now defined these acronyms in the abstract (see Line 54).

5. Line 104 - would be useful additional information to briefly summarise the reproducibility metrics.

We have now provided additional information regarding reproducibility metrics in the Methods section as follows:

Methods section, ‘Assessment of Pulmonary Hemodynamics’ sub-section, Line 166-168:

“Reproducibility metrics have been previously reported and demonstrated intra-reader coefficients of variation of less than 15% and correlations of greater than 0.93.”

6. Line 170 - I see later that the amount of follow-up was not the same for all patients (line 283). If follow-up differs substantially then might a time to event analysis be more suitable than logistic regression (which does not account for differential follow-up)?

Cross-sectional associations of prevalent left heart disease, pulmonary dysfunction, and VTE with abnormalities of PASP, PVR, and PAC were assessed using multivariable logistic regression. For the association of pulmonary vascular measures with incident HF, time to event analysis was performed using multivariable Cox proportional hazards models. We have now clarified this as follows:

Methods section, Statistical Methods sub-section, Line 235-239:

“Among ARIC participants attending Visit 5 and free of prevalent HF, the cross-sectional associations of prevalent left heart disease, pulmonary dysfunction, and VTE with abnormalities of PASP, PVR, and PAC were assessed using multivariable logistic regression with the pulmonary vascular measure as the outcome variable and adjusting for age, sex, race, and each of the potential etiologies.”

7. Line 178/results - Good that confidence intervals were provided for PARs - how were they calculated? How should a 'non-significant' PAR in table 2 be interpreted? I'm unsure marking as NS I informative.

Population attributable risk was estimated using methods appropriate for cohort and cross-sectional studies, with normalizing and variance-stabilizing transformation to define the corresponding confidence intervals [^[3]^,^[4]^].

8. Statistical methods: were covariates used in the multivariable models missing in a non-negligible proportion of patients? If so please justify the missing data assumptions made.

Covariates in multivariable models were age, sex, race, visit center, BMI, hypertension, diabetes, LVEF, LVMi, LAVi and E/e. None of the participants were missing for age, sex, race, visit center, hypertension, diabetes, and LVEF. 15 participants had missing BMI, 10 had missing LVMi, 17 had missing LAVi, and 8 had missing E/E’. As missing covariates were present in <1%, we considered this negligible.

9. Line 214-215: I would recommend providing confidence intervals rather than '+/-' values.

Standard deviations are removed and confidence intervals are now provided. Please see Line 276-278.

10. Table 2 - minor type but I assume PAR for prior VTE abnormal PASP should be (-0.2,3.0)

We apologize for this typographical error and have now corrected it. Please see revised Table 2 (Line 329).

**Reviewer #2:**

1. Age-appropriate reference limits for pulmonary vascular measures were defined in a subset of low risk participants free of prevalent cardiovascular disease or risk factors. In my opinion, it is crucial to explain why this subset was defined as reference. Why not to compare with other established cut-off values?

We appreciate the comments of the Reviewer. While established clinical cut points exist for PASP and PVR, these pulmonary vascular measures are known to change with age, and other characteristics such as BMI [^[5]^]. We therefore aimed to describe the limits of normal for our sample from the distribution in a sub-group known to be at a low risk of relevant CV events. Notably, while the resulting 90^th^ percentile limit for PASP was lower than the commonly employed clinical cut point, it agrees well with recent data from invasive RHC regarding the level of PA pressure at which risk of adverse CV events increases [^[6]^].

We have now repeated our primary analyses using a clinical cut-off value of 40 mmHg for PASP and 3.0 WU for PVR. As illustrated in Response Tables 1 and 2 (below), findings of cross-sectional correlates and associations with incident events were consistent with those of our primary analysis. Power was quite limited for associations with incident HFpEF or HFrEF.

We have now modified the Results section as follows:

Results section, “Associations of left heart disease, pulmonary dysfunction, and venous thromboembolism with pulmonary vasculature dysfunction” sub-section, Line 320-324:

“Similar findings were noted when using commonly employed cut-off values of 40 mmHg for PASP and 3.0 WU for PVR (S5 Table), and when further excluding an additional 196 participants with moderate or severe dyspnea and ESC HFA criterial for HFpEF (S6 Table).”

Results section, “Relationship of pulmonary vascular hemodynamics with NT-proBNP, RV function, and incident HF” sub-section, Line 375-381:

“Similar findings were also observed when modeling the pulmonary vascular measures as continuous variables (S8 Table), when using inverse probability weights (S9 Table), when further excluding an additional 196 participants with moderate or severe dyspnea and ESC HFA criteria for HFpEF (S10 Table), and when using cut-off values of 40 mmHg for PASP and 3.0 WU for PVR, although power was limited for associations with incident HFpEF and HFrEF (S11 Table).”

Response Table 1 was added as Supplemental Table 5. Response Table 2 was added as Supplemental Table 11.

Response Table 1. Prevalence of left heart dysfunction, pulmonary dysfunction and VTE, and their association with pulmonary vascular dysfunction. Abnormal pulmonary measures defined by established reference limits.

|  | N abnormal | OR (95%CI) | P value | PAR (95%) |
| --- | --- | --- | --- | --- |
| **Abnormal PASP** | Total n=2810 |  |  |  |
| LHD | 798 (28%) | 4.53 (2.71-7.58) | < 0.001 | 18.6 (12.0-24.7) |
| Pulmonary dysfunction | 994 (35%) | 2.37 (1.42-3.97) | 0.001 | 13.6 (4.6-21.8) |
| Prior VTE | 76 (3%) | 1.96 (0.67-5.79) | 0.22 | 0.9 (-0.8, 2.7) [NS] |
| **Abnormal PVR** | Total n=2798 |  |  |  |
| LHD | 793 (28%) | 2.89 (1.28-6.51) | 0.01 | 36.8 (1.62, 59.4) |
| Pulmonary dysfunction | 988 (35%) | 3.35 (1.32-8.51) | 0.01 | 52.9 (5.64, 76.5) |
| Prior VTE | 76 (3%) | 1.24 (0.15-10.0) | 0.84 | 0.7 (-6.97, 7.84) [NS] |

Legend: LHD, left heart disease; VTE, venous thromboembolism; PAR, population attributable risk; NS, not significant

Odds values and p-values are derived from multivariable logistic models containing age, sex, race, visit center LHD, pulmonary dysfunction, and prior VTE.

Response Table 2. Association of pulmonary hemodynamic measures based on established reference limits with incident HF or death post-Visit 5.

| Abnormal pulmonary vasculature measures | Clinical outcome | Number of events  Normal/Abnormal | Even rate  (per 100  person-years)  Normal/Abnormal | HR [95% CI] |
| --- | --- | --- | --- | --- |
| Total n=2810  Abnormal PASP 86 (3.1%) | HF | 142 / 12 | 0.98 / 3.14 | 2.20 [1.15-4.22] |
|  | HFrEF | 60 / 2 | 0.42 / 0.52 | 0.90 [0.21-3.88] |
|  | HFpEF | 61 / 7 | 0.42 / 1.83 | 2.39 [0.96-5.98] |
|  | HF or death | 407 / 32 | 2.81 / 8.35 | 2.55 [1.72-3.77] |
|  | HFrEF or death | 351 / 27 | 2.42 / 7.04 | 2.65 [1.73-4.06] |
|  | HFpEF or death | 357 / 30 | 2.46 / 7.83 | 2.74 [1.82-4.12] |
|  |  |  |  |  |
| Total n=2798  Abnormal PVR 30 (1.1%) | HF | 146 / 6 | 1.00 / 4.34 | 1.63 [0.66 -4.02] |
|  | HFrEF | 60 / 2 | 0.41 / 1.45 | 0.90 [0.19-4.25] |
|  | HFpEF | 64 / 2 | 0.43 / 1.45 | 1.10 [0.19-6.34] |
|  | HF or death | 420 / 10 | 2.90 / 7.23 | 1.58 [0.82-3.04] |
|  | HFrEF or death | 367/ 8 | 2.50 / 5.79 | 1.56 [0.76-3.22] |
|  | HFpEF or death | 376 / 8 | 2.56 / 5.79 | 1.55 [0.75-3.21] |

Legend: HF, heart failure; HFrEF, heart failure with reduced ejection fraction (LVEF <50%); HFpEF, heart failure with preserved ejection fraction (LVEF ≥50%). P-values were derived from multivariable Cox regression model adjusted for age, sex, race, visit center, BMI, hypertension, diabetes, LVEF, LAVi, LVMi and septal E/e’.

2. Clarify how left heart dysfunction was defined. It was present in 30% at baseline. Patients with LVEF<40% at baseline were excluded from the study?

Prevalent left heart dysfunction was defined as an LVEF <50% or echocardiographic evidence of elevated left atrial pressure based on left atrial enlargement (left atrial volume index >34 ml/m^2^) or elevated E/e’ ratio (average E/e’ >14) [^[7]^]. on Visit 5 echocardiogram. (Line 188-191) Patients with prevalent HF at Visit 5 were excluded from this analysis. However, LVEF alone was not an exclusion criterion. Of the 2,810 participants included in this analysis, 5 had an LVEF <40%.

3. Cox proportional hazards models were employed to assess the association of PASP, PVR and PAC with incident HF overall, incident HFrEF incident HFpEF, and the composite (HF/death). Please, clarify the length of follow-up, the number of events (persons/years) and the covariates include in all models.

The length of follow-up time was mean±SD of 5.3±1.2 years. The number of events and event rate in person-years for each outcome are now shown in Figure 4 and are provided in all relevant Supplemental Tables. Multivariable Cox proportional hazards models adjusted for age, sex, race, visit center, BMI, hypertension, diabetes, and LV measures (LVEF, LAVi, LVMi and septal E/E’). We now additionally state the follow-up time and adjustment variables in the Results section:

Results section, ‘Relationship of pulmonary vascular hemodynamics with NT-proBNP, RV function, and incident HF’ sub-section, Line 362-369:

“Total of 446 cases of death or heart failure admission occurred over a mean±SD follow-up of 5.3±1.2 years within range of 6.6 years, 161 participants developed incident HF (71 HFpEF, 65 HFrEF, 25 HF with unknown LVEF) and 344 died. Incident HF or death occurred in 446 (59 with incident HF prior to death: 24 HFpEF, 26 HFrEF, 9 HF with unknown LVEF). In models adjusted for age, sex, race, visit center, BMI, hypertension, diabetes, and LV measures (LVEF, LAVi, LVMI and septal E/E’), higher PASP, higher PVR, and lower PAC were each predictive of an increased risk of death or incident HF (Fig 3B, S7 Table [model 3]).”

4. The study sample had no prior HF (theoretically). However, this is a high-risk population for HF (76 years old, 82% prior hypertension, and 34% type 2 DM). I speculate a representative proportion of these patients may have unrecognized clinical HF at baseline. What was the distribution of NT-proBNP at baseline? This is a crucial point. Clinical HF was ruled out? How?

Along the same line, the authors showed a significant association between pulmonary vascular dysfunction (PASP, PVR, and PAC) with NT-proBNP at baseline. I speculate some of these patients may have clinical HF at baseline. How many of these patients fulfilled the criteria for diagnosing HF at baseline. Please, report. It should be also interesting to include NT-proBNP in all models, especially in those prognostic ones and the evaluate the prognostic ability of parameters of pulmonary dysfunction across the levels of NT-proBNP. This is issue should also be discussed more in depth.

We appreciate the concerns of the Reviewer. We have now revised our primary analysis to exclude all participants with an adjudicated HF hospitalization prior to Visit 5 (note: adjudication in ARIC began in 2005), hospitalization with a HF ICD code 428 in any position prior to 2005, or any self-report of HF or HF medication use on annual ARIC follow-up phone interviews prior to Visit 5. While we believe that this may have resulted in exclusion of several participants without HF (i.e. reduced specificity), this expansive definition of possible HF at Visit 5 should provide greater confidence that participants with prevalent HF were excluded (i.e. greater sensitivity). As a result of this change, 335 additional participants were excluded and the revised sample size for this analysis is 2,810. We have now revised all results accordingly throughout the manuscript. Importantly, however, the results remain consistent with those presented in our original submission.

To further assess the potential impact of including persons with undiagnosed HF, we performed an additional sensitivity analysis further excluding participants who self-reported presence of moderate or severe dyspnea at Visit 5 and who met HFpEF diagnostic criteria of the European Society of Cardiology Heart Failure Associated based on a score of ≥5 [^[8]^]. For this sensitivity analysis, additional 196 participants were excluded. As seen in Response Table 3 below, cross sectional associations of left heart dysfunction, pulmonary dysfunction, and VTE with pulmonary vascular measures were concordant with our primary analysis. The association of LHD with abnormal PAC was modestly attenuated. As seen in Response Table 4 below, the associations of pulmonary vascular measures with incident HF, HFpEF, and HFrEF were also consistent with our primary analysis. We have also provided a detailed description of the methodology for this sensitivity analysis in the Supplemental Materials. We have chosen to provide this as a sensitivity analysis (as opposed to the primary analysis) as we have previously shown that moderate or severe dyspnea is multifactorial in ARIC [^[9]^] and that cut-points for abnormal diastolic measures used in guidelines may not be appropriate for persons in late-life [^[10]^].

We have added the following text to the Results section:

Results section, “Associations of left heart disease, pulmonary dysfunction, and venous thromboembolism with pulmonary vasculature dysfunction” sub-section, Line 320-324:

“Similar findings were noted when using commonly employed cut-off values of 40 mmHg for PASP and 3.0 WU for PVR (S5 Table), and when further excluding an additional 196 participants with moderate or severe dyspnea and ESC HFA criterial for HFpEF (S6 Table).”

Results section, “Relationship of pulmonary vascular hemodynamics with NT-proBNP, RV function, and incident HF” sub-section, Line 375-381:

“Similar findings were also observed when modeling the pulmonary vascular measures as continuous variables (S8 Table), when using inverse probability weights (S9 Table), when further excluding an additional 196 participants with moderate or severe dyspnea and ESC HFA criteria for HFpEF (S10 Table), and when using cut-off values of 40 mmHg for PASP and 3.0 WU for PVR, although power was limited for associations with incident HFpEF and HFrEF (S11 Table).”

Response Table 3 was added as Supplemental Table 6. Response Table 4 was added as Supplemental Table 10.

Response Table 3. Prevalence of left heart dysfunction (LHD), pulmonary dysfunction, and venous thromboembolism (VTE), and their association with abnormal PASP, PVR, or PAC

|  | N abnormal | OR (95%CI) | P value | PAR (95%) |
| --- | --- | --- | --- | --- |
| **Abnormal PASP** | Total n=2614 |  |  |  |
| LHD | 696 (27%) | 2.15 (1.68-2.76) | <0.001 | 18.6 (12.0-24.7) |
| Pulmonary dysfunction | 885 (34%) | 1.46 (1.15-1.86) | 0.002 | 13.6 (4.6-21.8) |
| Prior VTE | 65 (2%) | 1.47 (0.76-2.83) | 0.25 | 0.9 (-0.8, 2.7) [NS] |
| **Abnormal PVR** | Total n=2604 |  |  |  |
| LHD | 692 (27%) | 1.06 (0.78-1.44) | 0.7 | 1.5 (-6.6, 9.0) |
| Pulmonary dysfunction | 881 (34%) | 1.33 (1.00-1.76) | 0.048 | 10.6 (-0.6, 20.6) [NS] |
| Prior VTE | 65 (2%) | 1.36 (0.62-3.00) | 0.44 | 0.8 (-1.4, 2.8) [NS] |
| **Abnormal PAC** | Total n=1995 |  |  |  |
| LHD | 553 (28%) | 1.36 (0.98-1.88) | 0.07 | 7.6 (-1.2-15.7) [NS] |
| Pulmonary dysfunction | 681 (34%) | 1.57 (1.15-2.15) | 0.004 | 17.5 (4.7-28.6) |
| Prior VTE | 54 (3%) | 0.55 (0.19-1.60) | 0.28 | -1.3 (-3.3-0.6) [NS] |

Legend: LHD, left heart disease; VTE, venous thromboembolism; PAR, population attributable risk; NS, not significant

Odds values and p-values are derived from multivariable logistic models containing age, sex, race, visit center LHD, pulmonary dysfunction, and prior VTE.

Response Table 4. Association of abnormalities of pulmonary vascular measures (PASP, PVR, PAC) with incident HF overall, incident HFpEF, incident HFrEF, or the composite of death with each of these. Models are adjusted for age, sex, race, visit center, BMI, hypertension, diabetes, LVEF, LAVi, LVMi, and septal E/e’.

| Abnormal pulmonary vasculature measures | Clinical outcome | Number of events  Normal/Abnormal | Even rate (per 100 person years)  Normal/Abnormal | HR [95% CI] |
| --- | --- | --- | --- | --- |
| Abnormal PASP Total n=2614 | HF | 83 / 40 | 0.71 / 1.77 | 1.86 [1.24-2.77] |
|  | HFrEF | 41 / 11 | 0.35 / 0.49 | 1.08 [0.54-2.17] |
|  | HFpEF | 31 / 19 | 0.27 / 0.84 | 2.18 [1.18-4.02] |
|  | HF or death | 277 / 93 | 2.37 / 4.11 | 1.44 [1.12-1.86] |
|  | HFrEF or death | 248 / 73 | 2.12 / 3.23 | 1.31 [0.99-1.73] |
|  | HFpEF or death | 245 / 80 | 2.10 / 3.54 | 1.39 [1.06-1.81] |
|  |  |  |  |  |
| Abnormal PVR Total n=2604 | HF | 104 / 19 | 0.84 / 1.23 | 1.09 [0.65 -1.81] |
|  | HFrEF | 45 / 7 | 0.37 / 0.45 | 0.66 [0.28-1.52] |
|  | HFpEF | 43 / 7 | 0.35 / 0.45 | 1.23 [0.54-2.84] |
|  | HF or death | 307 / 62 | 2.49 / 3.99 | 1.34 [1.00-1.78] |
|  | HFrEF or death | 265/ 55 | 2.15 / 3.54 | 1.34 [0.98-1.82] |
|  | HFpEF or death | 269 / 55 | 2.18 / 3.54 | 1.40 [1.03-1.90] |
|  |  |  |  |  |
| Abnormal PAC Total n=1995 | HF | 64 / 24 | 0.68/ 1.95 | 2.09 [1.26-3.45] |
|  | HFrEF | 20/ 12 | 0.21 / 0.97 | 3.04 [1.39-6.66] |
|  | HFpEF | 31 / 7 | 0.33 / 0.57 | 1.51 [0.63-3.64] |
|  | HF or death | 216 / 61 | 2.29 / 4.93 | 1.57 [1.15-2.13] |
|  | HFrEF or death | 186 / 52 | 1.97 / 4.20 | 1.49 [ 1.07-2.08] |
|  | HFpEF or death | 197 / 48 | 2.09 / 3.88 | 1.35 [0.96-1.90] |

Legend: HF, heart failure; HFrEF, heart failure with reduced ejection fraction (LVEF <50%); HFpEF, heart failure with preserved ejection fraction (LVEF ≥50%). P-values were derived from multivariable Cox regression model adjusted for age, sex, race, visit center, BMI, hypertension, diabetes, LVEF, LAVi, LVMi and septal E/e’.

As requested by the Reviewer, we have also performed additional analyses of the associations of abnormalities in pulmonary vascular measures (PASP, PVR, PAC) with incident HF overall, incident HFpEF, incident HFrEF, or the composite of death in multivariable Cox proportional hazard models adjusting for log transformed NT-proBNP in addition to demographics (age, sex, race, visit center, BMI), co-morbidities (hypertension and diabetes), and LV measures (LVEF, LAVi, LVMi and septal E/E’) (Response Table 5). These findings are concordant with those of our primary analysis. We also assessed for effect modification of log transformed NT-proBNP and on the associations of each pulmonary vasculature measure with incident HF using multiplicative interaction terms. No interactions were detected (all interaction p values >0.10).

Response Table 5. Associations of abnormalities of pulmonary vascular measures (PASP, PVR, PAC) with incident HF overall, incident HFpEF, incident HFrEF, or the composite of death with each of these in a model with further adjustment of log transformed NT-proBNP.

| Abnormal pulmonary vasculature measures | Clinical outcome | Number of events  Normal/Abnormal | Even rate (per 100 person years)  Normal/Abnormal | HR [95% CI] |
| --- | --- | --- | --- | --- |
| Abnormal PASP Total n=2810 | HF | 96 / 58 | 0.78 / 2.25 | 1.62 [1.13-2.32] |
|  | HFrEF | 45 / 17 | 0.37 / 0.66 | 1.00 [0.54-1.13] |
|  | HFpEF | 39/ 29 | 0.32 / 1.12 | 1.77 [1.04-3.02] |
|  | HF or death | 313 / 126 | 2.55 / 4.88 | 1.31 [1.05-1.65] |
|  | HFrEF or death | 277 / 101 | 2.25 / 3.91 | 1.23 [0.96-1.59] |
|  | HFpEF or death | 277 / 110 | 2.25 / 4.26 | 1.29 [1.01-1.65] |
|  |  |  |  |  |
| Abnormal PVR Toal n=2798 | HF | 130 / 22 | 0.99 / 1.32 | 0.63 [0.38 -1.04] |
|  | HFrEF | 53 / 9 | 0.40 / 0.54 | 0.27 [0.11-0.65] |
|  | HFpEF | 58 /8 | 0.44 / 0.48 | 0.74 [0.33-1.64] |
|  | HF or death | 365 / 71 | 2.78 / 4.23 | 1.02 [0.77-1.35] |
|  | HFrEF or death | 311/ 64 | 2.37 / 3.81 | 1.05 [0.77-1.41] |
|  | HFpEF or death | 321 / 63 | 2.44 / 3.75 | 1.10 [0.82-2.54] |
|  |  |  |  |  |
| Abnormal PAC Total n=2149 | HF | 76 / 36 | 0.76 / 2.57 | 1.70 [1.09-2.66] |
|  | HFrEF | 24/ 17 | 0.24 / 1.21 | 2.43 [1.20-4.93] |
|  | HFpEF | 37 / 14 | 0.37 / 1.00 | 1.44 [0.69-2.98] |
|  | HF or death | 246 / 85 | 2.46 / 6.05 | 1.44[1.09-1.90] |
|  | HFrEF or death | 210 / 73 | 2.10 / 5.19 | 1.42 [1.05-1.91] |
|  | HFpEF or death | 224 / 70 | 2.24 / 4.98 | 1.31 [0.97-1.77] |

5. The article is difficult to follow. There is a too much data/comparisons.

We have now attempted to simplify the data presented by removing additional details of cross-sectional associations presented in the Results ‘Associations of left heart disease, pulmonary dysfunction, and venous thromboembolism with pulmonary vasculature dysfunction’ sub-section, removing 4 supplemental tables (Former S5 through S8 Tables), and removing and one supplemental Figure (Former S4 Figure). We believe this has allowed us to make the manuscript more interpretable without compromising any of the key study findings.

**Reviewer #3:**

1. Methods: authors should consider addition of well-established risk factors for HF and PH to multivariable-adjusted models, including BMI, DM, HTN, which were not included - these may act as potential confounders given their associations with both PH and HF.

As requested by the Reviewer, we have now included BMI, hypertension and diabetes as adjustment variables in multivariable logistic and Cox proportional hazard models throughout the manuscript. Adjustment variables for multivariable models now include age, sex, race, visit center, BMI, hypertension, diabetes, LVEF, LAVi, LVMi and septal E/E’. The results of analyses with the addition of these adjustment covariates are similar to those of our original analysis.

2. Results: Authors define abnormal PASP, PVR, and PAC cut-points using reference limits. It is important to acknowledge that these reference limits represent lower cut-points when compared with accepted clinical measures. For example, PASP >=40 is usually what prompts further evaluation for PH (vs PASP deemed abnormal at >=32 here). With this definition of abnormal, it appears that more than half of individuals deemed to have "abnormal PVR" do not have abnormal PASP (Figure 1A) - is mean PAP elevated in those cases or how can abnormal PVR in absence of PAP elevation be understood?

Please refer to our response to Reviewer 2, Comment 1. Mean PAP was calculated in our study based on the tricuspid regurgitation spectral Doppler velocity-time integral. The mean±SD for mean PAP was 17.1±4.4 mmHg for abnormal PVR and 15.1±3.3 mmHg for normal PVR (p<0.001). The prevalence of abnormal mean PAP (using our reference limit derived from the low risk sub-group) among those with abnormal PVR was 38%.

3. Results: In S11 Table, none of the measures (PASP, PVR, PAC, when analyzed as continuous variables) were associated with HFrEF - yet when presented as dichotomous variables, PASP and PAC are associated. Can authors comment on this discrepancy? Is there a non-constant HR across the range of PASP?

In models adjusted for demographics, BMI, hypertension, diabetes, and LV measures, PASP was not associated with incident HFrEF when modeled as a dichotomous variable (Figure 4) or as a continuous variable (S8 Table). However, as noted by the Reviewer, PAC was significantly associated with incident HFrEF when modeled as a dichotomous variable but not as a continuous variable. We use restricted cubic splines to describe the continuous association between PAC and incidence of HFrEF, and to assess for a potential non-linear association. The number of knots was selected to minimize the model AIC with 3 to 5 knots tested. Spline models adjusted for demographics, BMI, hypertension, diabetes, and LV measures. The model with 3 knots demonstrated the smallest AIC (Response Figure). The p-value for non-linearity in this model was 0.07. The incidence of HFrEF appears to increase at values of PAC below 3 ml/mmHg, which may help explain the observed discrepancy between analyses with PAC modeled dichotomously compared to continuously.

Response Figure. Association between continuous PAC and incident HFrEF.

4. Differential prediction of HFpEF vs HFrEF: "abnormal PASP was predictive of both incident HFpEF and HFrEF, with a higher magnitude of effect noted for incident HFpEF" - did authors formally test for difference in HR? The number of outcomes here are small and I would encourage authors not to place too much emphasis on differential associations with HF subtypes in both the abstract and discussion

We agree with the Reviewer. We have now modified the Abstract, Results and Discussion sections to place less emphasis on the differential associations of PASP and PAC in particular with HF subtypes.

5. Discussion: authors suggest that pulmonary hypertension leads to development of HFpEF / HF - could it be that HF was just undiagnosed at the time of PV assessment and the two co-exist or PH is a manifestation of HFpEF (how much time between echo and HF diagnosis)? How many individuals may meet echo/NT-proBNP criteria for HFpEF at the time of echo? Would be careful with wording - authors did not diagnose pulmonary hypertension - the cut-points used in this paper are derived reference limits but are lower compared with clinical definitions of PH.

We appreciate the Reviewer’s concern. Please refer to our response to Reviewer 2, Comment #4. The median time duration between the day of the echocardiography and diagnosis of HF was 3.7 [IQR .2.4-4.5] years. We have now replaced ‘pulmonary hypertension’ with ‘elevated pulmonary pressure’ or ‘abnormal pulmonary pressure’ throughout the manuscript. Please refer to Lines 54, 447, 510.

**Reviewer #4:**

1) A large majority of the study participants were female. While this is a corrective to the often male-dominated clinical studies, it is not reflective of the general US and other populations worldwide. Conclusions drawn should be cautiously interpreted.

We have now added the following text to the Limitations Paragraph of the Discussion:

Discussion, Line 499-500:

“The majority of participants in this study were women, which may limit the generalizability of these findings.”

2) Why was a single measure of RAP used when determining PASP? This is not in keeping with previous and current ASE guidelines.

Unfortunately, measurement of inferior vena cava diameter, and assessment of inferior vena cava collapsibility, was not part of the echocardiographic protocol in ARIC Visit 5. Therefore, a RAP of 5 mmHg was uniformly applied. Of note, this approach of adding a uniform value for RAP is frequently used in this field of this study [^[11]^,^[12]^,^[13]^,^[14]^].

3) Please provide more information on HFpEF outcomes.

a. how was LVEF adjudicated in these cases?

LVEF from the time of hospitalization was ascertained by trained chart abstractors as the lowest assessed LVEF at the time of the HF admission, preferably by transthoracic echocardiography. When no LVEF assessment was performed or available from the heart failure admission, the most recent and lowest LVEF available within the prior to 6 months was used if there was no intercurrent myocardial infarction. The HF hospital record abstraction form can be found at the ARIC website [^[15]^]. We have now added this information to the Supplement Material Methods section.

b. were 2016 ESC criteria for HFpEF satisfied in the majority of cases?

No, quantitative data necessary to assess the 2016 ESC criteria were not uniformly available. Incident HFpEF was adjudicated based on HF signs and symptoms and an LVEF >50%. Importantly, this approach is concordant with the majority of epidemiologic and clinical trial studies in this field [^[16]^].

4) The authors tout the value of characterizing multiple measures of pulmonary vascular function, other than PASP. They should highlight also the limitations of noninvasive determination of PAC and PVC. PAC is indirectly derived from PASP, as well as other measures that can be technically challenging to accurately ascertain. PVR is also dependent on TR velocity while RVOT outflow velocity curve is not infrequently technically inadequate. This may be why the number of subjects with measurable PAC and PVR (detailed in see Fig 3) are lower, and in the case of PAC, much lower than subjects with measurable PASP. While this is a large study, the smaller number of outcomes arising from the reduced sample size with measurable PAC does increase the potential for statistical over-fitting.

We fully agree with the Reviewer. We have now added the following text to the Limitations paragraph of the Discussion to highlight these issues:

Discussion, Line 492-495:

“Nonetheless, the use of echocardiography-based measures could result in appreciable misclassification. PVR and PAC are highly dependent on TR velocity, and the limited number participants with measurable values for PAC in particular may have led to over-fitting of some models.”

5) Did PAC, when analyzed dichotomously, have incremental value in predicting HFpEF over PASP? With respect to outcomes summarized graphically in Fig 3, was PAC still predictive of HFpEF after correction for PASP?

In models adjusting for demographics and abnormal PASP (Model 1 in Response Table 6 below), abnormal PAC was no longer associated with incident HF overall (HR 1.15 [95% CI 0.93-1.43], p=0.20) but was associated with incident HFrEF (1.56 [1.05-2.30], p=0.03). This association was no longer significant after further adjustment for BMI, hypertension, diabetes, and LV measures (LVEF, LVMI, LAVi and septal E/E’) (Model 2 in Response Table 6). Associations with the composite of HF or death, HFrEF or death, and HFpEF or death persisted in both models.

Response Table 6. Association between abnormal PAC and HF incidence.

|  |  | Model 1 | |  | Model 2 | |
| --- | --- | --- | --- | --- | --- | --- |
| Clinical outcome |  | HR [95% CI] | p-value |  | HR [95% CI] | p-value |
| HF |  | 1.15 [0.93-1.43] | 0.20 |  | 1.04 [0.84-1.27] | 0.74 |
| HFrEF |  | 1.56 [1.05-2.30] | 0.026 |  | 1.20 [0.83-1.75] | 0.34 |
| HFpEF |  | 0.98 [0.73-1.31] | 0.88 |  | 0.97 [0.72-1.29] | 0.82 |
| HF or death |  | 1.23 [1.08-1.40] | 0.001 |  | 1.15 [1.01-1.31] | 0.03 |
| HFrEF or death |  | 1.33 [1.15-1.53] | < 0.001 |  | 1.22 [1.06-1.41] | 0.007 |
| HFpEF or death |  | 1.23 [1.07-1.40] | 0.003 |  | 1.16 [1.01-1.33] | 0.031 |

Model 1: adjusted for age, sex, race, visit center, PASP

Model 2: Model 1 covariates, BMI, hypertension, diabetes, and LV measures (LVEF, LVMi, LAVi and septal E/E’)

6) Line 397: "Both high PVR and lower PAC were associated with worse RV systolic function, while PASP - within the range represented in this study sample - was not associated with RVFAC."

The authors will be well aware that PASP is dependent on RV function, and by itself does not provide information on right ventricular-pulmonary arterial coupling.

RV-PA coupling has been shown in several publications to predict death and recurrent HF in subjects with HF, and the risk of RV failure in patients with pulmonary hypertension.

Since the authors have data on RV function, i.e. fractional area changes and tricuspid annulus systolic velocity, it would be instructive to evaluate if a measure of RV-PA coupling would be even more useful in predicting adverse HF and other outcomes.

We have now performed additional analyses assessing the prognostic value of RVFAC/PASP ratio as a measure of RV-PA coupling. Lower RVFAC/PASP ratio was associated with greater risk of incident HF overall and incident HFpEF, but not incident HFrEF (Response Table 7). It was also associated with greater risk of death or HF overall, death or HFpEF, and death or HFrEF. These results are consistent with those in a previously publication from the ARIC cohort in a smaller subset of participants with 3D assessment of RV function (i.e. RVEF) [^[17]^]. In that study, RVEF/PASP ratio was assessed as a measure of RV-PA coupling.

Response Table 7. Association between PASP/RVFAC and incident HF, per 1-unit change.

|  |  | Model 1 | |  | Model 2 | |  | Model 3 | |
| --- | --- | --- | --- | --- | --- | --- | --- | --- | --- |
| Total n = 2674 | Number of events | HR [95% CI] | p-value |  | HR [95% CI] | p-value |  | HR [95% CI] | p-value |
| HF | 144 | 1.56 [1.30-1.87] | < 0.001 |  | 1.36 [1.14-1.63] | 0.001 |  | 1.38 [1.15-1.66] | 0.001 |
| HFrEF | 56 | 1.13 [0.86-1.48] | 0.37 |  | 1.05 [0.79-1.38] | 0.75 |  | 1.04 [0.78-1.38] | 0.79 |
| HFpEF | 65 | 1.80 [1.37-2.37] | < 0.001 |  | 1.52 [1.16-2.00] | 0.003 |  | 1.53 [1.16-2.01] | 0.003 |
| HF or death | 403 | 1.25 [1.13-1.39] | < 0.001 |  | 1.17 [1.06-1.30] | 0.003 |  | 1.21 [1.08-1.34] | 0.001 |
| HFrEF or death | 345 | 1.19 [1.07-1.33] | 0.002 |  | 1.13 [1.01-1.26] | 0.039 |  | 1.17 [1.04-1.31] | 0.008 |
| HFpEF or death | 357 | 1.24 [1.11-1.38] | < 0.001 |  | 1.17 [1.05-1.31] | 0.005 |  | 1.20 [1.08-1.35] | 0.001 |

Model 1: adjusted for age, sex, race, visit center

Model 2: Model 1 covariates, BMI, hypertension, diabetes

Model 3: Model 2 covariates, LV measures (LVEF, LVMi, LAVi and septal E/E’)

7. Minor concerns

a. Line 321: "...emerging as an important contributor to HF general..."

Should read "HF in general"

Thank you. We have now corrected this typographical error.

b. Line 336: "...abnormalities of distinct dimensions of pulmonary vascular function..."

What does this mean?

We meant to indicate that different pulmonary vascular measures may differentially predict incident HF phenotype. It is now rephrased as follows:

Line 411-413; “These findings suggest that impairments in pulmonary vascular dysfunction antecede clinical HF, and abnormalities of different measures of pulmonary vasculature may differentially predict incident HF phenotype.”

c. Line 351: "(RC time)"

Please qualify all abbreviations.

We have now revised the manuscript to define all abbreviations. Please refer to line 426.

**Academic Editor**

1. I agree with them in that the independent temporal relationship between pulmonary vascular dysfunction and heart failure needs further exploration. Indeed, the authors themselves show a similar association in the other direction: people with HF had higher rates of abnormal pulmonary dysfunction. So, whether the new finding is due to measurement error (undetected or incipient HF at baseline), uncontrolled confounding (the robustness of the findings are borderline), or a circular relationship between HF and vascular resistance needs further exploration.

We appreciate the comment of the Academic Editor. Please refer to our response to Reviewer 2, Comment 4.

2. I also agree with one of the reviewers that the sheer number of tables, figures and numbers is a bit overwhelming and suggest that they simplify the paper to make it more accessible to the general medical audience.

We have now attempted to simplify the data presented by removing additional details of cross-sectional associations presented in the Results ‘Associations of left heart disease, pulmonary dysfunction, and venous thromboembolism with pulmonary vasculature dysfunction’ sub-section, removing 4 supplemental tables (Former S5 through S8 Tables), and removing and one supplemental Figure (Former S4 Figure). We believe this has allowed us to make the manuscript more interpretable without compromising any of the key study findings.

References Cited

1. The Atherosclerosis Risk in Communities (ARIC) Study: design and objectives. The ARIC investigators. Am J Epidemiol. 1989 Apr;129(4):687-702.
2. Shah AM, Cheng S, Skali H, Wu J, Mangion JR, Kitzman D, Matsushita K, Konety S, Butler KR, Fox ER, Cook N, Ni H, Coresh J, Mosley TH, Heiss G, Folsom AR, Solomon SD. et al. Rationale and design of a multicenter echocardiographic study to assess the relationship between cardiac structure and function and heart failure risk in a biracial cohort of community-dwelling elderly persons: the Atherosclerosis Risk in Communities study. Circ Cardiovasc Imaging. 2014 Jan;7(1):173-81.
3. Greenland, S., and K. Drescher. 1993. Maximum likelihood estimation of the attributable fraction from logistic models. Biometrics 49: 865-872.
4. Newson, R.B., 2013. Attributable and unattributable risks and fractions and other scenario comparisons. The Stata Journal, 13(4), pp.672-698.
5. Brendan M. McQuillan, Michael H. Picard, Marcia Leavitt, and Arthur E. Weyman. Clinical Correlates and Reference Intervals for Pulmonary Artery Systolic Pressure Among Echocardiographically Normal Subjects. Circulation. 2001;104:2797–2802.
6. Maron BA, Hess E, Maddox TM, Opotowsky AR, Tedford RJ, Lahm T, et al. Association of Borderline Pulmonary Hypertension With Mortality and Hospitalization in a Large Patient Cohort: Insights From the Veterans Affairs Clinical Assessment, Reporting, and Tracking Program. Circulation. 2016;133(13):1240-1248.
7. Nagueh SF, Smiseth OA, Appleton CP, Byrd BF 3rd, Dokainish H, Edvardsen T, et al. Recommendations for the Evaluation of Left Ventricular Diastolic Function by Echocardiography: An Update from the American Society of Echocardiography and the European Association of Cardiovascular Imaging. Eur Heart J Cardiovasc Imaging. 2016;17(12):1321-1360.
8. Pieske B, Tschöpe C, de Boer RA, Fraser AG, Anker SD, Donal E, Edelmann F, Fu M, Guazzi M, Lam CSP, Lancellotti P, Melenovsky V, Morris DA, Nagel E, Pieske-Kraigher E, Ponikowski P, Solomon SD, Vasan RS, Rutten FH, Voors AA, Ruschitzka F, Paulus WJ, Seferovic P, Filippatos G. How to diagnose heart failure with preserved ejection fraction: the HFA-PEFF diagnostic algorithm: a consensus recommendation from the Heart Failure Association (HFA) of the European Society of Cardiology (ESC). Eur Heart J. 2019 Oct 21;40(40):3297-3317.
9. Ramalho SHR, Santos M, Claggett B, Matsushita K, Kitzman DW, Loehr L, Solomon SD, Skali H, Shah AM. Association of Undifferentiated Dyspnea in Late Life With Cardiovascular and Noncardiovascular Dysfunction: A Cross-sectional Analysis From the ARIC Study. *JAMA Netw Open* 2019;2:e195321.
10. Shah AM, Claggett B, Kitzman D, Biering-Sørensen T, Jensen JS, Cheng S, Matsushita K, Konety S, Folsom AR, Mosley TH, Wright J, Heiss G, Solomon SD. Contemporary assessment of left ventricular diastolic function in older adults: the Atherosclerosis Risk in Communities Study. *Circulation* 2017;135:426-39
11. Zile MR, Gottdiener JS, Hetzel SJ, McMurray JJ, Komajda M, McKelvie R, Baicu CF, Massie BM, Carson PE; I-PRESERVE Investigators. Prevalence and significance of alterations in cardiac structure and function in patients with heart failure and a preserved ejection fraction. Circulation. 2011 Dec 6;124(23):2491-501.
12. Amil M. Shah, MD MPH, Sanjiv J. Shah, MD, Inder S. Anand, MD, Nancy K. Sweitzer, MD PhD, Eileen O’Meara, MD, John F. Heitner, MD, George Sopko, MD MPH, Guichu Li, PhD RDCS, Susan F. Assmann, PhD, Sonja M. McKinlay, PhD, Bertram Pitt, MD, Marc A. Pfeffer, MD PhD, Scott D. Solomon, MD, and for the TOPCAT Investigators. Cardiac Structure and Function in Heart Failure with Preserved Ejection Fraction: Baseline Findings from the Echocardiographic Study of the Treatment Of Preserved Cardiac Function Heart Failure with an Aldosterone Antagonist (TOPCAT) Trial. Circ Heart Fail. 2014; 7(5): 740–751.
13. Shah AM, Cikes M, Prasad N, Li G, Getchevski S, Claggett B, Rizkala A, Lukashevich I, O'Meara E, Ryan JJ, Shah SJ, Mullens W, Zile MR, Lam CSP, McMurray JJV, Solomon SD; PARAGON-HF Investigators. Echocardiographic Features of Patients With Heart Failure and Preserved Left Ventricular Ejection Fraction. J Am Coll Cardiol. 2019 Dec 10;74(23):2858-2873.
14. Choudhary G, Jankowich M, Wu WC. Elevated pulmonary artery systolic pressure predicts heart failure admissions in African Americans: Jackson Heart Study. Circ Heart Fail. 2014 Jul;7(4):558-64.
15. https://sites.cscc.unc.edu/aric/desc_pub
16. Marc A. Pfeffer, Amil M. Shah, Barry A. Borlaug. Heart Failure With Preserved Ejection Fraction

    In Perspective. Circ Res. 2019;124:1598-1617.
17. Nochioka K, Querejeta Roca G, Claggett B, Biering-Sørensen T, Matsushita K, Hung C-L, Solomon SD, Kitzman D, Shah AM. Right ventricular function, right ventricular-pulmonary artery coupling, and heart failure risk in the community: the Atherosclerosis Risk in Communities (ARIC) study. *JAMA Cardiol* 2018;3:939-48.
