# Supplementary material for: Pulmonary vascular dysfunction among people aged over 65 years in the community in the Atherosclerosis Risk In Communities (ARIC) Study: A cross-sectional analysis
Source: PLoS Med. 2020 Oct 15;17(10):e1003361. doi: 10.1371/journal.pmed.1003361 (PMC7561082; doi:10.1371/journal.pmed.1003361)
Supplement: Attachment — Submitted filename: Response to Reviewers_PMEDICINE-D-19-04388_0817.docx [file pmed.1003361.s019.docx]

**Response to Editor and Reviewers**

Thank you so much for your acceptance of my manuscript entitled, ‘Pulmonary vascular dysfunction among people aged over 65 years in the community in the Atherosclerosis Risk In Communities (ARIC) Study: a cross-sectional analysis.”

Followings are our additional response to valuable comments and suggestions from the Editor and Reviewers.

**Request from the editors;**

**1. Please provide full stops after reference brackets.**

We have provided full stops (.) after reference brackets.

**2. Line 469 should say “heightened” perhaps**

We have changed “greater risk” to “heightened risk”. Thank you for your suggestion.

**3. Data- please provide an accession number or direct link to the dataset. If there are restrictions on data sharing, please mention these in the data availability statement, providing reasons for these.**

The ARIC datasets are available through BioLINCC, with appropriate study approvals consistent with NIH policies. Data request forms through BioLINCC can be accessed at <https://biolincc.nhlbi.nih.gov/studies/aric/>. We have now added this information to the data availability statement.

**4. Author summary-could you reword “time to event analysis” using more accessible language? Please also remove "Non technical" from the author summary heading.**

We have revised the Author summary as follows: “We performed a cross-sectional analysis and an analysis of associations with subsequent occurrence of clinical heart failure”.

We also have removed “Non technical” from the author summary heading.

**5. Please provide the STROBE checklist as a separate supplementary file and include a callout to this file in the methods. Please also use paragraphs and sections instead of page numbers as these are likely to change.**

We have modified our STROBE checklist as a separate file and used paragraphs and sections instead of page numbers. The STROBE checklist is specifically called out in the method section.

**6. Please provide a direct link to the interviewer administered questionnaires used in ARIC and cite this where the questionnaires are mentioned in the methods section.**

Questionnaires used in ARIC and for this manuscript can be obtained at <https://sites.cscc.unc.edu/aric/cohort-forms-forms> and we have now provided this link in the Method section.

**Comments from the Reviewer #4**:

**1. Methods section, 'Assessment of Pulmonary Hemodynamics' sub-section, Line 166-168:**

**For reproducibility metrics of pulmonary vascular variables, coefficients of variation and correlations are not meaningful. Please provide the intraclass correlation coefficients for the raw measurements.**

The number we have shown in the revised manuscript was indeed an intraclass correlation coefficients for the raw measurements. We clarified in the manuscript text that it refers to an intraclass correlation coefficients.

**2. Results section, "Associations of left heart disease, pulmonary dysfunction, and venous thromboembolism with pulmonary vasculature dysfunction" sub-section, Line 320-324 and**

**Results section, "Relationship of pulmonary vascular hemodynamics with NT-proBNP, RV function, and incident HF" sub-section, Line 375-381: Why were only those 196 participants with moderate or severe dyspnea and ESC HFA criteria for HFpEF excluded? Why not also those with LVEF <50% using the 2016 ESC criteria for HFrEF and HFmrEF?**

After excluding the 196 participants with moderate or severe dyspnea and who met ESC HFA criteria for HFpEF those patients, there were 31 participants with LVEF <50%. Only one of these participants also had moderate or severe dyspnea. We therefore believe it is unlikely for this to have influenced our findings. However, given the Reviewer’s concern regarding participants with mid-ranged LVEF, we have now further excluded these 31 participants with LVEF <50%. A total of 227 participants are now excluded in this sensitivity analysis, the results of which are provided in updated Tables S6 and S0. The results do not differ appreciably from the original analysis and also from previous sensitivity analysis.

**3. Results section, 'Relationship of pulmonary vascular hemodynamics with NT-proBNP, RV function, and incident HF' sub-section, Line 362-369: "...mean±SD follow-up of 5.3±1.2 years within range of 6.6 years..." does not look right.**

The corrected range was 6.5 years. We have now corrected this in the manuscript text.

**4. Table S11 appears to be truncated - there are no data on PAC.**

Table S11 provides associations of pulmonary hemodynamic measures with incident HF or death when dichotomized using established reference limits. As established reference limits for PAC are not defined, we did not include PAC in this table.

**5. Minor concerns:
a. Even rate should be Event rate**

We have now made this correction.

**b. PAR (95%) should be PAR (95% CI)**
We have now made this correction.

**c. There are several spelling errors.**
We have now proof-read the Supplemental materials document closely and corrected all spelling errors.
